# Supplementary material for: Serial femtosecond crystallography on in vivo-grown crystals drives elucidation of mosquitocidal Cyt1Aa bioactivation cascade
Source: Nat Commun. 2020 Mar 2;11:1153. doi: 10.1038/s41467-020-14894-w (PMC7052140; doi:10.1038/s41467-020-14894-w)
Supplement: Supplementary file 1 — Supplementary Information [file 41467_2020_14894_MOESM1_ESM.pdf]

Supplementary Information

Serial femtosecond crystallography on *in vivo*-grown crystals drives elucidation of mosquitocidal Cyt1Aa bioactivation cascade

Guillaume Tetreau, Anne-Sophie Banneville, Elena A. Andreeva, Aaron S. Brewster, *et al*.

This PDF file contains:

- Supplementary Note 1
- Supplementary Figures 1-16
- Supplementary Tables 1-4

Supplementary Note 1

A difference-density based mutation strategy

The Fo$^{'DTT'}$-Fo$^{'pH7'}$ map indicates that soaking with DTT results in rupture of the disulfide bridge (strong negative peak on the C7-Sγ-Sγ-C7 bond) and, consequently, in a relaxation of the N-terminal propeptide resulting in an expansion at crystal packing interface #3 where the N-terminal propeptide (notably D11), the C-terminal end of β6, and the N-terminal ends of β5 (notably Q168) and β7 interact (Fig. 2 and Supplementary Fig. 3 and Supplementary Table 1). Marked by a positive peak between D11 and Q168, this expansion seemingly impacts the hydrophobic core of the protein, with negative peaks observed on the aromatic side chains of F147, Y171 and Y173. Refinement of the 'DTT' structure confirms that DTT has no further effect on the protoxin structure at pH 7 (Fig. 2b and Supplementary Figs. 2 and 4).

Features in the Fo$^{'pH10'}$-Fo$^{'pH7'}$ map differ, with strong positive and negative peaks at the DS interface and no peaks on disulfide bridge atoms (Fig. 2b and Supplementary Fig. 4). A positive peak is again seen between D11 and Q168 at crystal packing interface #3, reminiscent of the observation made in the Fo$^{'DTT'}$-Fo$^{'pH7'}$ map. Refinement of the 'pH10' structure confirms changes in interactions at the DS interface (Fig. 2 and Supplementary Figs. 2 and 4), with loss of the bifurcated intermolecular H-bond between the side chains of E32 (β1-β2 loop) and E45 (β2-αA loop) from facing monomers, and of the intramolecular H-bond tethering the αC/αD region (Q138) to the tip of β2 (E45). As a result, in each monomer, strands β1, β2 and the C-terminal propeptide draw away from strand β3, at the opposite end of the β-sheet, but also from the αC, αD and αE helices, which together cover the hydrophobic face of the β-sheet (Supplementary Fig. 2). To the contrary, the αA/αB face of the β-sheet is not affected by pH elevation, in line with Fo$^{'pH10'}$-Fo$^{'pH7'}$ map observations.

We challenged by mutagenesis the role of residues pinpointed by the strongest peaks in the Fo$^{'DTT'}$-Fo$^{'pH7'}$ and Fo$^{'pH10'}$-Fo$^{'pH7'}$ maps, hypothesizing that they would be central to crystal formation and dissolution, and possibly function (Fig. 3). We first probed the role of disulfide-bridge chaining by expressing a C7S mutant. The C7S crystals are spherical and significantly smaller than the WT crystals, but they are produced at a similar yield and diffract to a similar resolution (2.0 vs. 1.85 Å, respectively, for similarly sized datasets), revealing a structure nearly indiscernible from the 'DTT' structure (Table 1, Figs. 2b and 3 and Supplementary Figs. 2 and 4). Thus, cysteine chaining of WT Cyt1Aa monomers is nonessential for the production of diffraction-grade crystals and could occur after *in vivo* crystallization has completed in *Bti* cells. As a control for the unicity of the role of C7, we also mutated the second cysteine of Cyt1Aa, viz. C190, introducing a C190V mutation with the aim to not interfere with the hydrophobic environment present at the αC/αD interface with the β-sheet (Fig. 1e and Supplementary Fig. 2). C190V crystals are significantly smaller than the WT but production

yields are not affected, suggesting a minor impact of the mutation on protoxin folding and *in vivo* crystallization (Fig. 3).

E32 and E45 were identified as the cornerstone residues controlling the pH sensitivity of the DS interface (Fig. 2b). With the aim of rendering pH-insensitive the H-bonds that these residues contribute at the DS interface, we mutated both residues into glutamines (E32Q, E45Q). The E45Q mutation was also intended to strengthen the tether between the αC/αD region (Q138) and the β-sheet, allowing us to test the hypothesis that opening of this interface is involved in function. E45Q crystals are significantly larger than the WT crystals, while E32Q crystals are produced in higher abundance (Fig. 3). Thus, both mutations, intended to stabilize the DS interface, positively impact crystal growth, albeit in a different fashion.

Our observation that interface 3 changes structurally in response to reducing agent and pH prompted us to test the role of D11 and Q168 in conferring these sensitivities. A D11N mutation was introduced to decrease the pH sensitivity of this interface, and a reverse Q168E mutation to increase it. Crystals of D11N are significantly larger and quasi-cylindrical in shape, evincing that the mutation favours crystal growth (Fig. 3). Crystals of Q168E are marquise-shaped and produced in reduced amounts (Fig. 3), highlighting the negative effect of the mutation on *in vivo* crystallization; the side chain nitrogen of Q168 is indeed involved in two H-bonds with acidic oxygen atoms at interface #3 (Fig. 2B and Supplementary Figs. 2, 3b,c and 4). Last, we introduced a Y171F mutation, attempting to verify whether the pH-sensitive H-bond between the β5 residue hydroxyl and T146(O) (pKr of tyrosine is 10.5) at the tip of αD plays a role in protein folding or alkaline activation. Indeed, packing relaxation at interface #3 was evident as disorder on the hydrophobic αC/αD face of the β-sheet (Fig. 2b). Both the production yield and the crystal size are negatively impacted (Fig. 3), suggesting that this H-bond plays an important role before crystallization – possibly during folding.

Supplementary Figures 1-16:

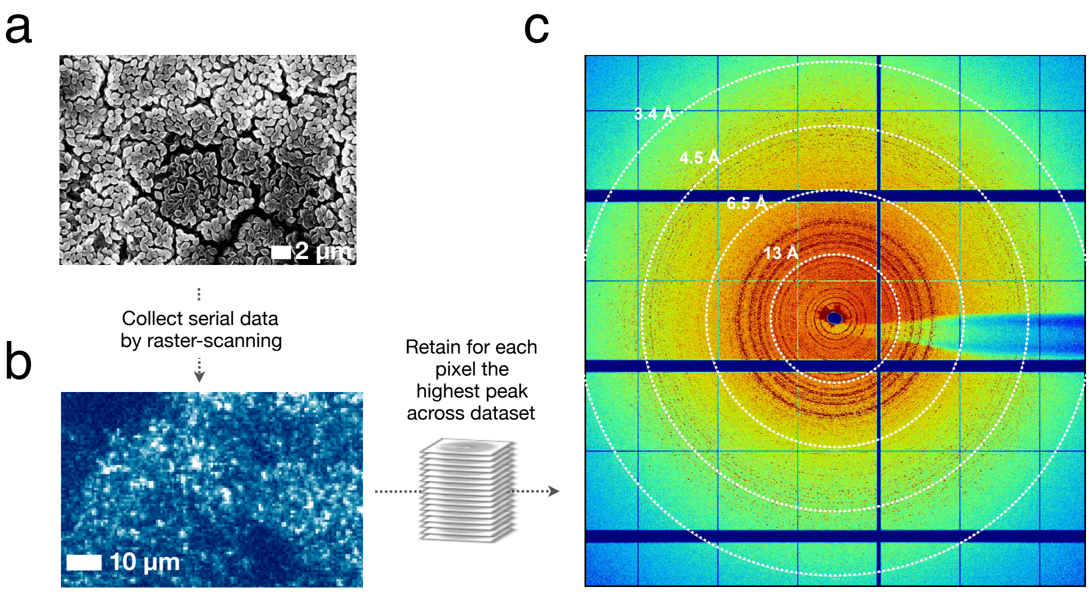

Supplementary Fig. 1. Serial synchrotron crystallography allowed assessing diffraction quality prior to XFEL experiments. **a**, Cyt1Aa nanocrystals were deposited between two silicon wafers and flash-cooled in a gaseous nitrogen stream at 100 K. **b**, Diffraction data were collected serially by raster-scanning with a sub-micron X-ray beam (0.7 µm FWHM), with 80 ms exposure per pattern and 10 µm horizontal and vertical spacing between X-ray shots. In this panel, each pixel represents a diffraction pattern, with the colouring intensity scaling to the summed intensity of Bragg peaks. The figure offers a X-ray microscopic image of the sandwiched crystal suspension presented to the X-ray beam. **c**, Powder rings extend to 4.0 Å resolution on the maximum projection of ~1 million diffraction patterns collected at 100 K using raster-scanning serial synchrotron crystallography at the (sub)-microfocus ESRF-ID13 beamline.

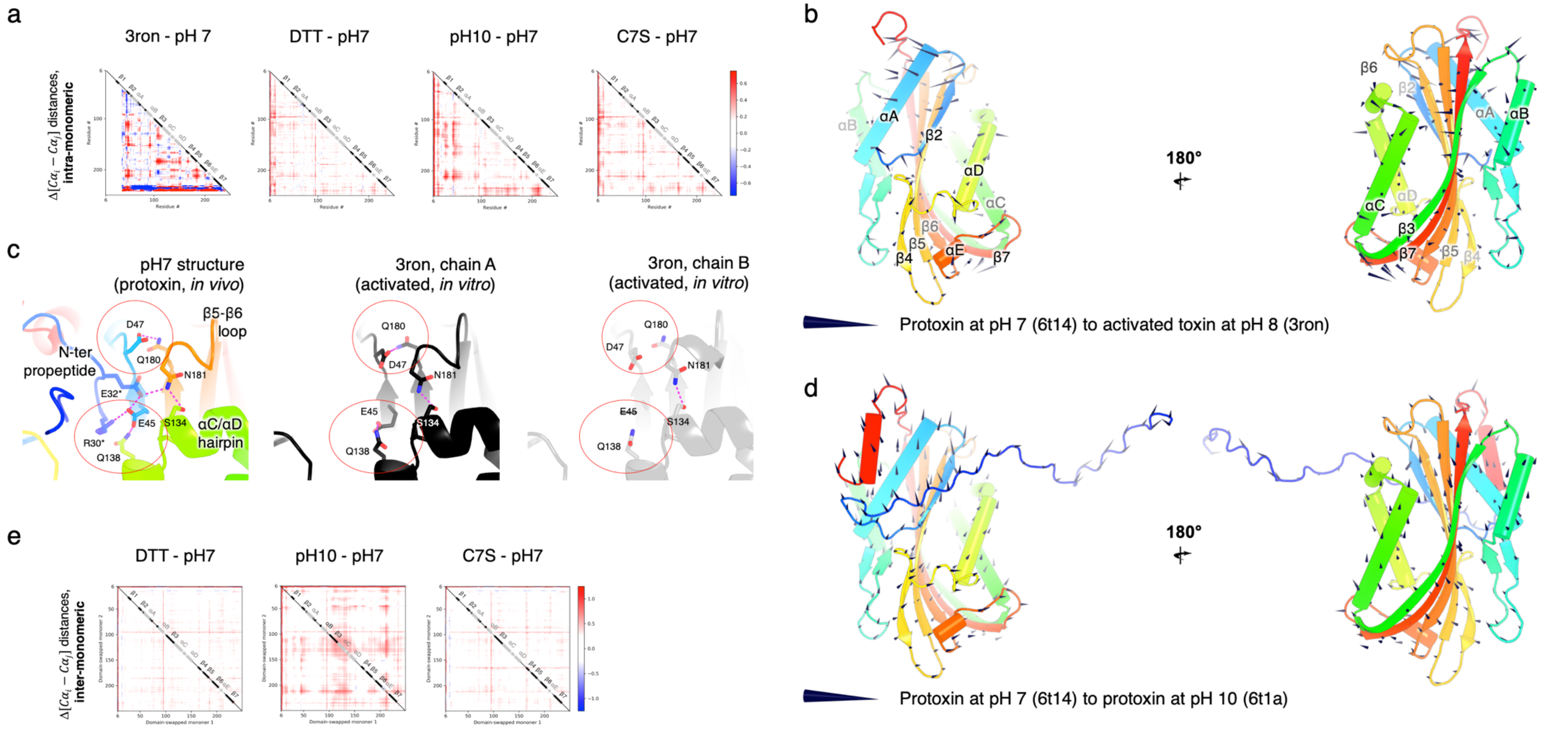

Supplementary Fig. 2. Structural differences between the Cyt1Aa protoxin and toxin structure. a, From left to right, intramolecular difference distance matrices highlight changes in Cα-Cα distances with respect to the protoxin structure ('pH7' structure) upon proteolytic activation (3ron), soaking with DTT ('DTT' structure), pH elevation ('pH10' structure) or C7S mutation ('C7S' structure), respectively. **b**, Porcupine plot highlighting the displacement of Cα atoms between the protoxin ('pH7' structure) and toxin structures (3ron). Each arrow shows the direction along which a Cα atom moves upon proteolytic activation, with the length of the arrow scaling to the amplitude of the motion; here, arrows are 10 times larger than the distance travelled by Cα atoms. No data is shown for residues 234-238, which concentrate the largest conformational changes and whose Cα-arrows would dominate the plot, rendering visualisation of other motions difficult. **c**, The H-bond between E138 and E45, which tethers the αC/αD hairpin to the β-sheet (left panel; same colouring as in **b**), is affected by proteolytic activation, being either preserved in a different conformation (chain A of the asymmetric unit; middle panel) or absent (no density for E45 in chain B of the asymmetric unit; right panel) in the activated toxin structure (3ron). Red cycles highlight the side chain conformational changes of interest. **d**, Porcupine plot highlighting the displacement of Cα atoms between the protoxin structure at pH7 ('pH7' structure) and at pH 10 ('pH10 structure'). Each arrow shows the direction along which a Cα atom moves upon pH elevation, with the length of the arrow scaling to the amplitude of the motion; here, arrows are 10 times larger than the distance travelled by Cα atoms. **e**, From left to right, intermolecular difference distance matrices highlight changes in Cα-Cα distances between monomers in the domain-swapped dimer upon soaking with DTT, pH elevation or C7S mutation, respectively.

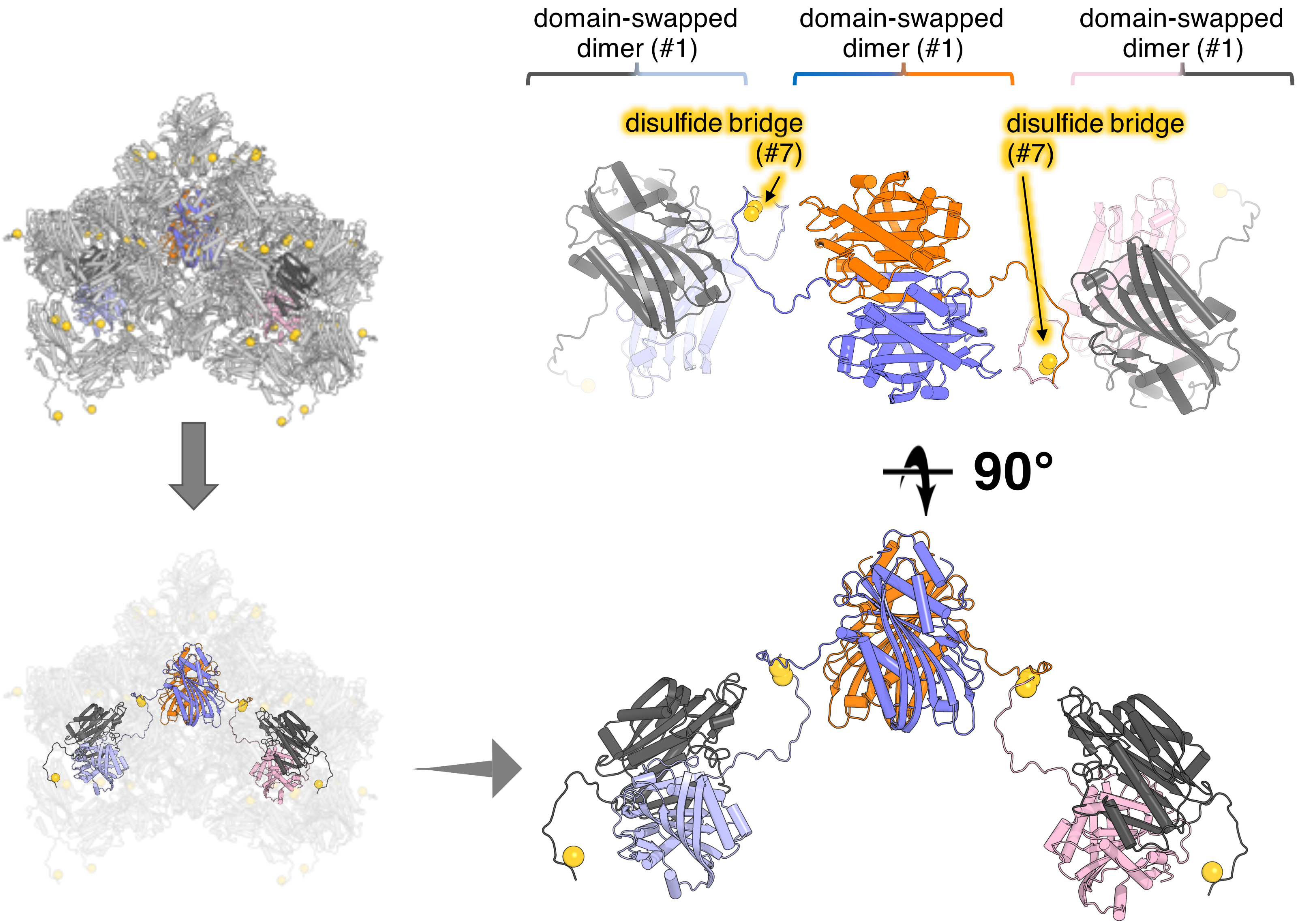

Supplementary Fig. 3. The natural Cyt1Aa crystals are highly packed. a, Natural Cyt1Aa crystals display a solvent content of ~33% and feature chains of domain-swapped (DS) dimers associated by disulfide bridges. For more details on crystal packing interfaces, please see Supplementary Table 1.

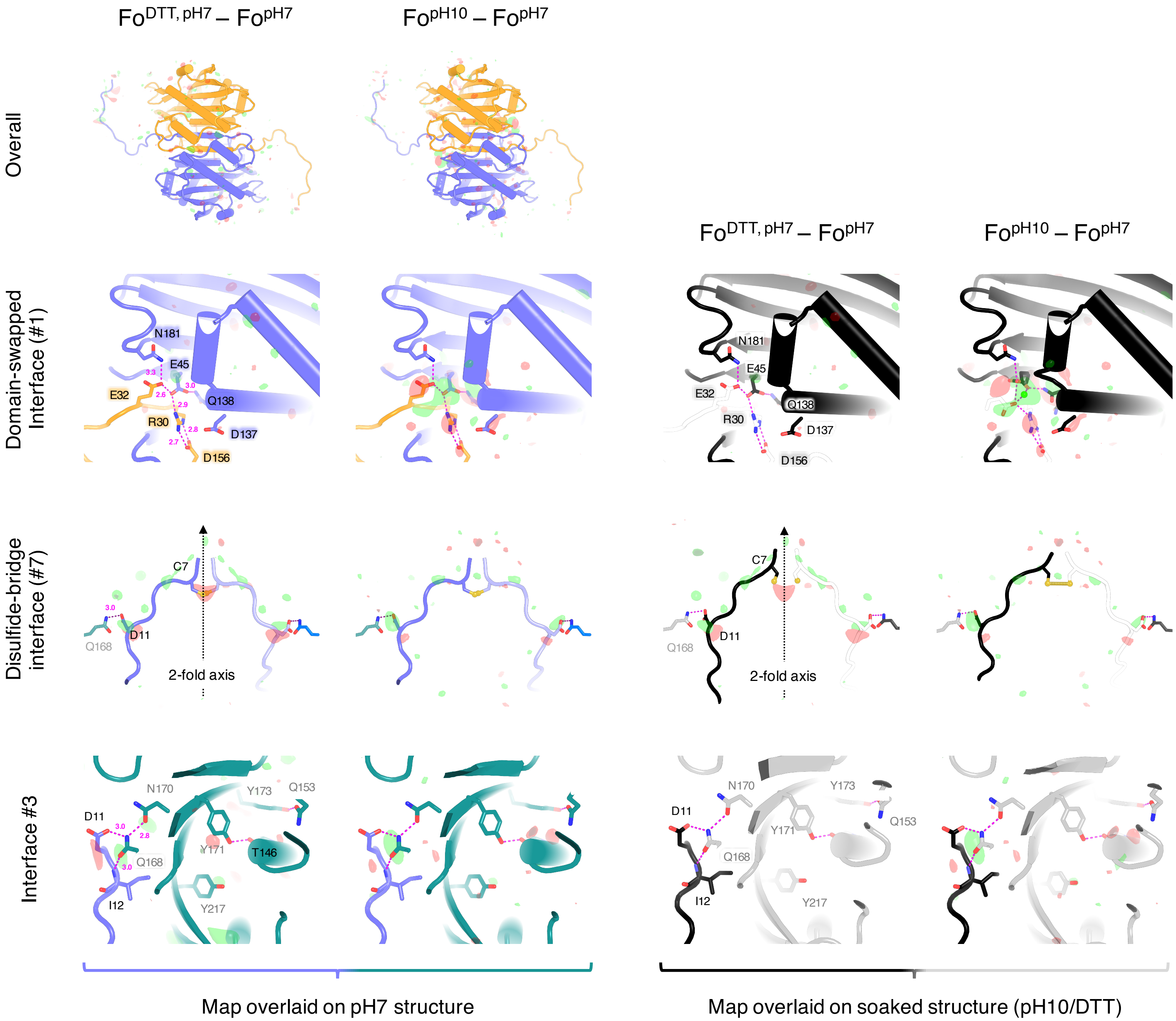

Supplementary Fig. 4. Fourier difference maps shed light on the crystal dissolution mechanism. Fourier difference maps computed between datasets, and phased by the pH7 structure, highlight the most striking conformational changes upon DTT soak ($Fo^{'DTT'}$-$Fo^{'pH7'}$ map) and pH elevation ($Fo^{'pH10'}$-$Fo^{'pH7'}$ map). These are overlaid on the pH7 protoxin structure, shown as an orange/slate-coloured ribbon, in the two left panels; and on the DTT/pH10 protoxin structure, shown as a black/white-coloured ribbon, in the two right panels. Symmetry related molecules are all coloured differently, with each molecule having the same colour coding in all panels. From top to bottom, the figure shows the maps contoured at ± 3 sigma around the full DS dimer, at the DS and cysteine interfaces, and at crystal packing interface 3, respectively, with positive and negative peaks shown in green and red.

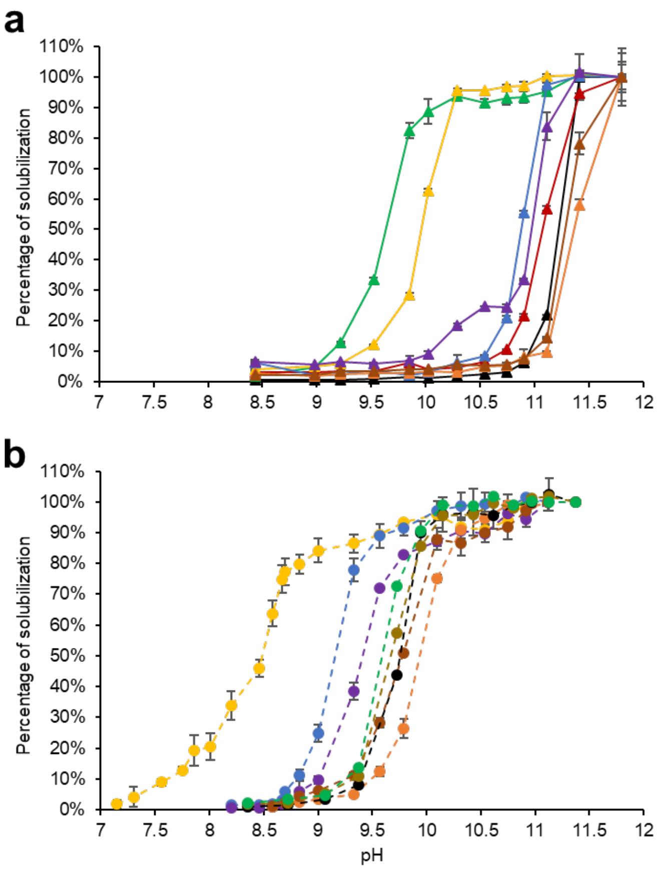

Supplementary Fig. 5. The pH-sensitivity of Cyt1Aa crystals depends on the redox condition and can be influenced by single atom substitutions. The concentration of solubilized toxin was measured following resuspension at different pHs in the absence (**a**) or presence (**b**) of DTT. Crystals of WT (black), C7S (green), C190V (red), Y171F (purple), D11N (blue), Q168E (yellow), E32Q (brown) and E45Q (orange) Cyt1Aa were assayed. Data are presented as percentage of solubilization relative to solubilization at the highest pH tested (mean ± SD). Source data are provided as a Source Data file.

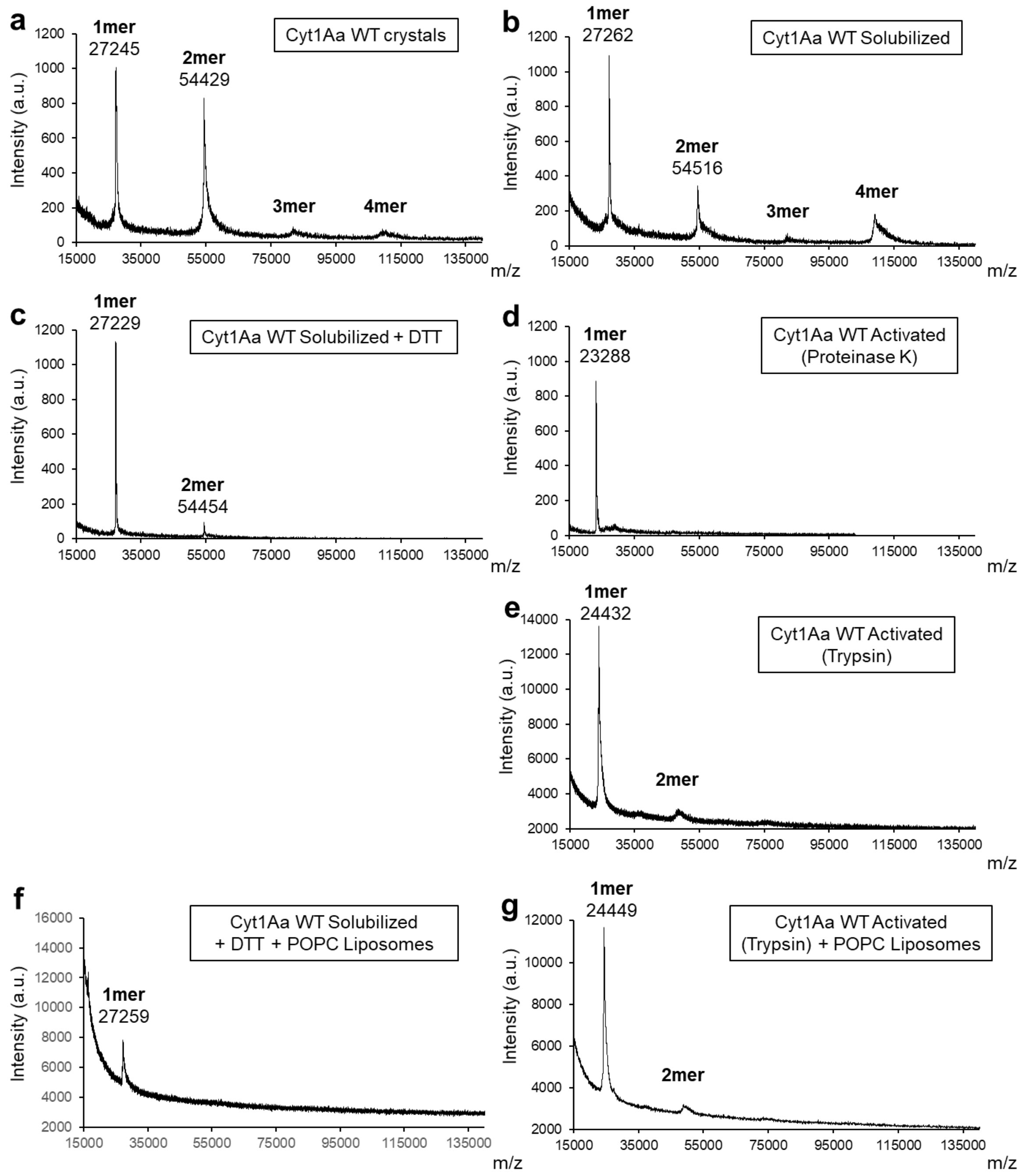

Supplementary Fig. 6. MALDI-ToF mass spectrometry confirms the SDS-PAGE characterization of the species released upon solubilization of Cyt1Aa crystals. In the various panels, we report spectra for different Cyt1Aa samples, prepared as indicated in the overlaid box. The observed *m/z* values for the monomeric $[M+H]^+$ (all spectra) and dimeric $[2M+H]^+$ (spectra **a** to **c**) species are indicated. The calculated (theoretical) average mass for Cyt1Aa protoxin is 27341.17 Da. The data were collected either directly from crystals (**a**) or from crystals solubilized in 0.1 M Na_2CO_3 buffer at pH 11.8 in absence (**b**) or presence of DTT (**c**). Solubilized protoxin activated into toxin by the use of proteinase K (**d**) or trypsin (**e**) were also analysed. The data collected on MBO (**f, g**) show that these are formed from fully-sized monomers of the protoxin (**f**) and proteolytically-activated toxin (**g**), respectively.

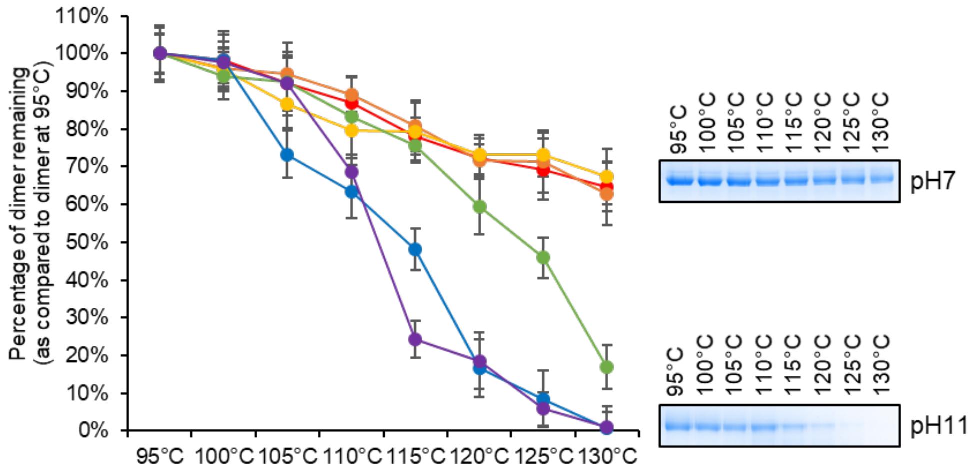

Supplementary Fig. 7. The high-temperature stability profile of the Cyt1Aa dimer at various pH is suggestive of a covalent disulfide link between monomers. The stability of the WT Cyt1Aa dimer was tested in two different forms: within crystals at pH7 (red) and as a soluble dimer at pH 7 (brown), 8 (yellow), 9 (green), 10 (blue) and 11 (purple), following crystals dissolution at pH 11.8 in the absence of DTT and subsequent pH equilibration. Samples were heated to 95-130°C for 5 min prior to loading on a 12 % SDS-PAGE gel. The amount of remaining dimer was evaluated by measuring the intensity of the corresponding band. For each pH, the ratio between the amount of dimer released at the various temperatures and that released at 95°C is reported. Each temperature was tested in triplicate. Data are represented as mean ± SE. Source data and uncropped images are provided as a Source Data file.

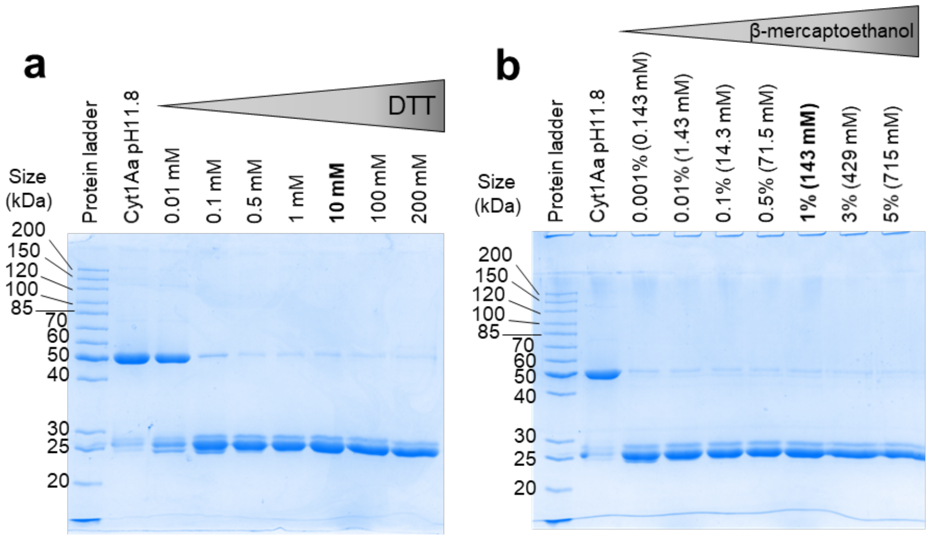

Supplementary Fig. 8. The Cyt1Aa dimer released upon dissolution of crystals at alkaline pH is highly sensitive to reducing agents. Crystals of WT Cyt1Aa were solubilized at pH 11.8, in absence or presence of increasing concentrations of DTT (**a**) or β-mercaptoethanol (**b**), prior to loading on 12% SDS-PAGE gel. The DTT and β-mercaptoethanol concentrations used in others assays are highlighted in bold. Uncropped images are provided as a Source Data file.

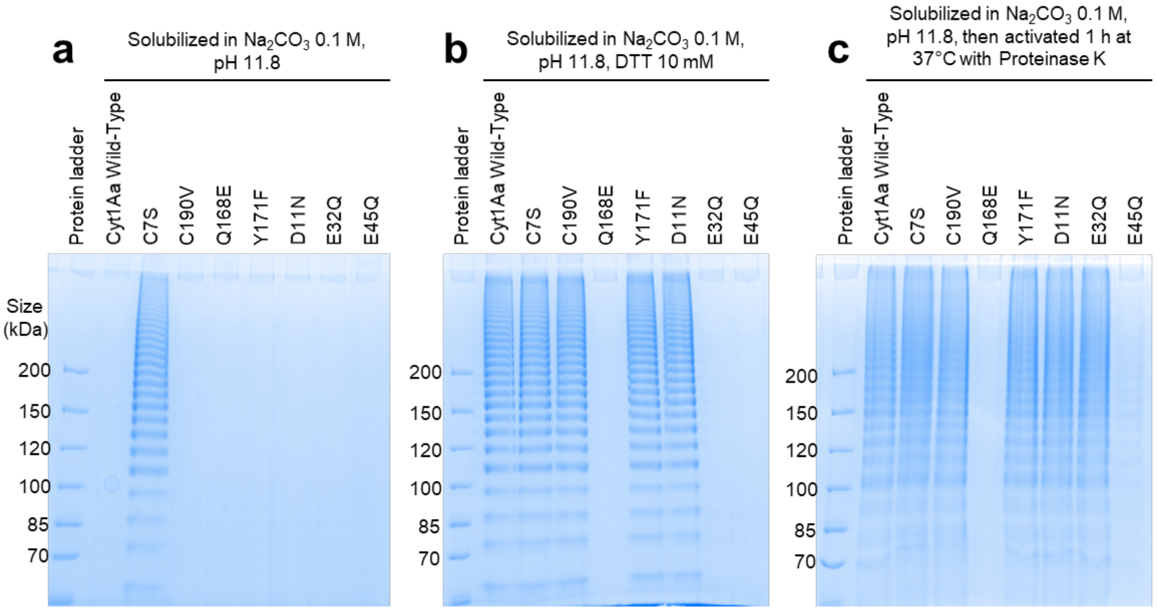

Supplementary Fig. 9. Formation of Cyt1Aa MBO is conditioned by rupture of the disulfide bridge, but can occur in presence of the N-terminal propeptide and be controlled by single atom substitutions. MBO formation by the disulfide-bridged dimer (**a**; obtained by solubilization of crystals at pH 11.8 in absence of DTT), the protoxin monomer (**b**; obtained by solubilization of crystals at pH 11.8 in presence of DTT) and the proteolytically-activated toxin (**c**; obtained by solubilization of crystals at pH 11.8 followed by 1 h incubation with proteinase K at 37°C) upon contact with 100 nm radius liposomes was assessed for WT and mutant Cyt1Aa by electrophoresis on a 6% SDS-PAGE gel. In the absence of DTT or proteolytic activation (**a**), only the C7S mutant, released from crystals as a protoxin monomer, is able to form MBO upon contact with liposomes. Rupture of the disulfide bridge by addition of DTT (**b**) rescues MBO formation by WT Cyt1Aa and mutants other than the E32Q, E45Q and Q168E mutants. Removal of the propeptides (**c**) elicits MBO formation by the E32Q and E45Q mutants, showing that stabilization of the DS dimer inhibits MBO formation. Contrastingly, the Q168E mutant remains unable to form MBO even after removal of the propeptide, suggesting that the mutation blocks a crucial step in the interaction of Cyt1Aa with the lipid membrane. Uncropped images are provided as a Source Data file.

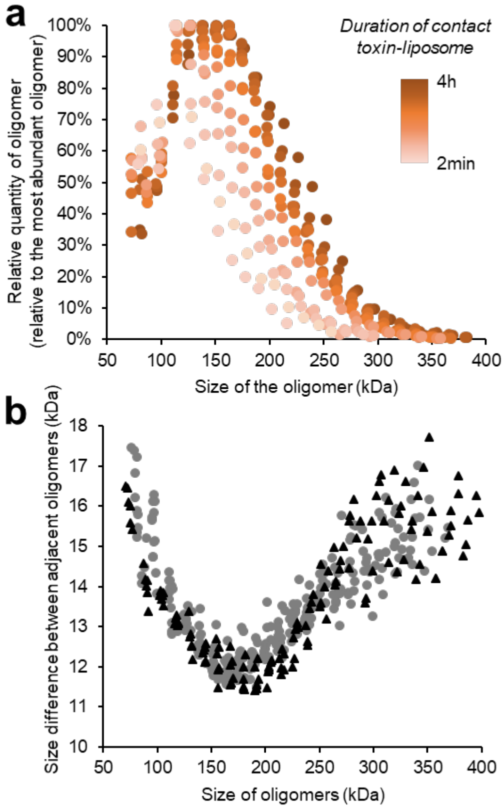

Supplementary Fig. 10. Prolonged incubation of Cyt1Aa with liposomes results in larger MBO, but their size and spacing in size in SDS-PAGE gels cannot be trusted to estimate their actual mass. Crystals of Cyt1Aa WT were solubilized at pH 11.8 in presence of DTT and incubated with liposomes (100 nm radius) for times ranging from 2 min (light orange) to 4 h (dark brown) prior to loading on a 6% SDS-PAGE gel stained with Instant*Blue.* ImageJ software v1.51k was used to measure the intensity of each oligomer band. **a**, MBO appear on 6% SDS-PAGE gels as a ladder of bands whose maximal size increases as function of time. Assuming that each band corresponds to a full Cyt1Aa monomer increment, as inferred from mass spectrometry measurements which showed that the building block of MBO is a fully-sized monomer of the protoxin (Supplementary Fig. 6f) or proteolytically-activated toxin (Supplementary Fig. 6g), the minimal and maximal sizes observed on the gels after 4 hours are indicative of at least trimers (first band migrating at ~79 and ~72 kDa for the protoxin and activated toxin, respectively) to 26-mers (~0.6 MDa). **b**, Estimation of the molecular mass of oligomers by performing a regression based on the molecular mass markers nonetheless indicates a maximum size of 0.35 MDa and suggests a spacing of ~14 kDa between the bands (after 2 hours of incubation) that varies with the size of the oligomers (values were extracted from six and nine different gels for protoxin (black triangle) and activated toxin (gray circles), respectively). This observation highlights the importance of mass spectrometry in complement of electrophoresis to cross-validate observations and identify experimental biases; it indeed could have been interpreted as a second proteolytic step taking place post-insertion of Cyt1Aa in the membrane. Source data are provided as a Source Data file.

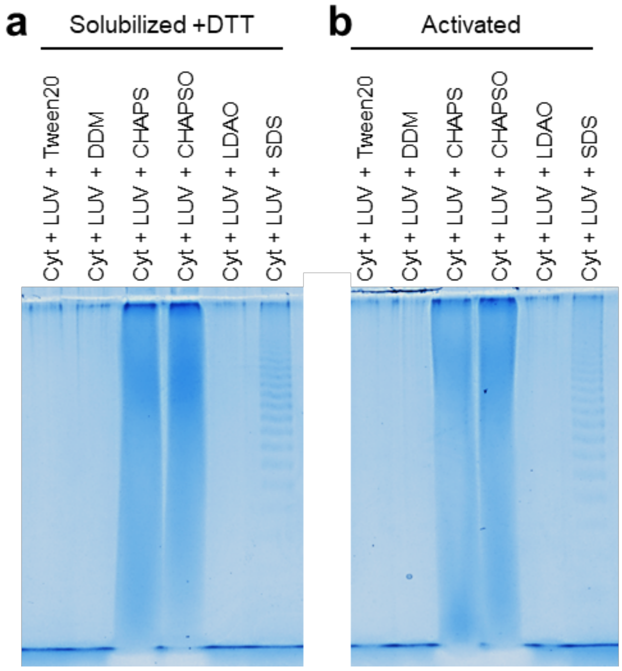

Supplementary Fig. 11. Detergents other than SDS are unable to induce the stepwise dissociation of MBO. WT monomeric Cyt1Aa protoxin (**a**; crystals solubilized at pH 11.8, in presence of DTT) and activated toxin (**b**; crystals solubilized at pH 11.8 and incubated for 1 h with proteinase K at 37°C) were incubated with liposomes (100 nm radius) for 1 hour. Various detergents were added to different aliquots of the sample which were then loaded on a 6% native gel stained with Instant*Blue* after completion of electrophoresis. The ladder profile, characteristic of the step-wise dissociation of MBO as they migrate through SDS-PAGE gels, is only visible when SDS is the detergent used to treat the MBO prior to their loading on the native PAGE gels. Other detergents tested are unable to induce this step-wise dissociation, and MBO therefore do not penetrate the gel. These observations highlight the unique ability of SDS in enabling the breakdown and identification of Cyt1Aa MBO. They also suggest that non SDS-treated MBO are likely larger than ~0.6 MDa, *i.e.* the largest size that could be estimated on our 6% acrylamide gels. Note that contrast and luminosity were adjusted to facilitate the visualization of the ladder-like pattern. Uncropped images without contrast optimization are provided as a Source Data file.

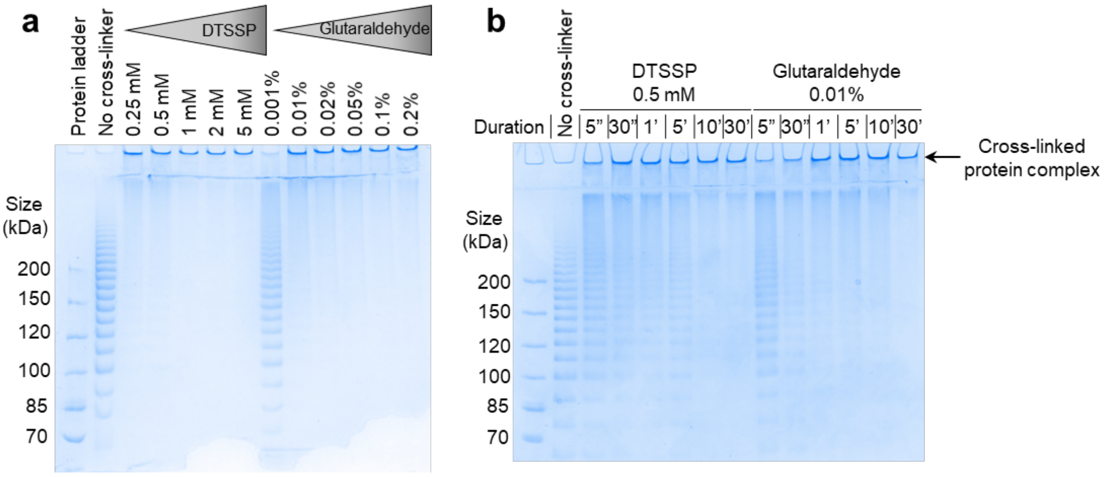

Supplementary Fig. 12. Cross-linking of Cyt1Aa MBO suggests that they are larger than visible on SDS-PAGE gels. WT Cyt1Aa MBO were prepared by addition of the activated toxin (crystals solubilized at pH 11.8 and incubated for 1 h with proteinase K at 37°C) to liposomes (100 nm), and cross-linked by incubation with DTSSP or glutaraldehyde at different concentrations and for different times. **a**, Two hours incubation with the cross-linkers at various concentrations allowed identifying the minimal concentration required to cross-link MBO while still allowing penetration into the 6% SDS-PAGE gels, viz. 0.5 mM DTSSP and 0.01 % glutaraldehyde. **b**, We attempted to capture the kinetics of MBO formation by incubating these for various times (from 5 s to 30 min) with 0.5 mM DTSSP or 0.01 % glutaraldehyde. After 5 min incubation, the MBO do not undergo stepwise dissociation in the presence of SDS, indicating that they have been efficiently cross-linked, but they also do not penetrate the stacking gel anymore, suggesting that they are far larger than ~0.6 MDa. Uncropped images are provided as a Source Data file.

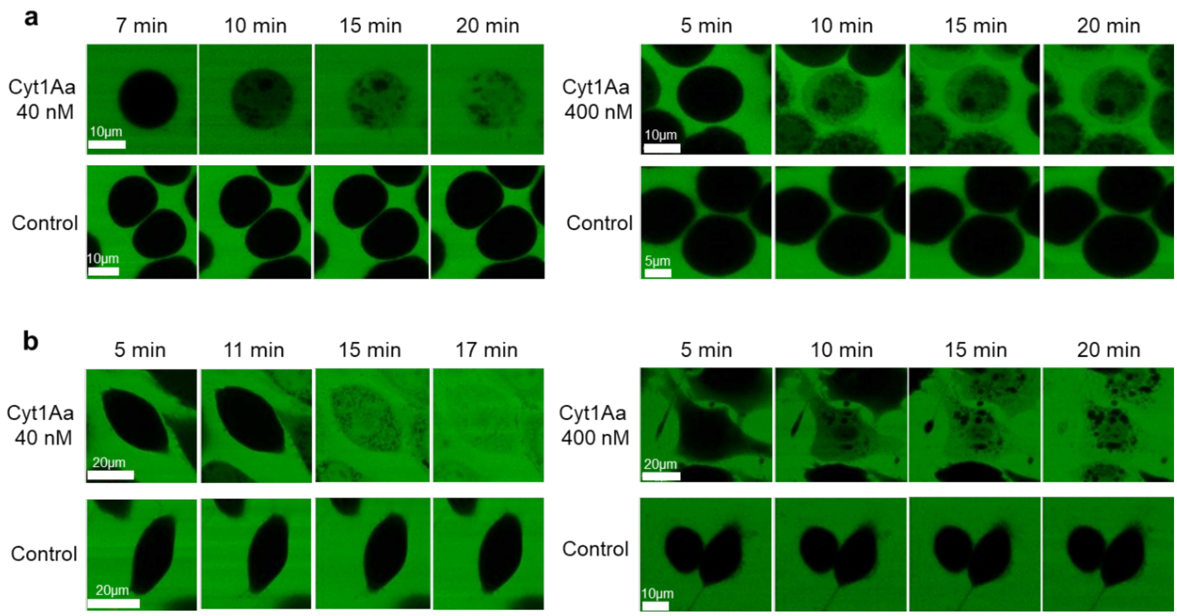

Supplementary Fig. 13. Time-series confocal fluorescence micrographs reveal the process of membrane permeabilization. Insect Sf21 (**a**) and mammalian NIH fibroblast (**b**) cells were co-exposed to 1.4 nm FITC-dextran and to either Cyt1Aa toxin (at 40 or 400 nM) or to buffer (control). Micrographs suggest a cytotoxicity mechanism whereby Cyt1Aa forms large holes in cell membranes upon insertion in these, enabling dextran beads to enter the cytoplasm within minutes at sub-lethal concentrations. Note that contrast and luminosity were adjusted for better visualization as in Fig. 5b-c.

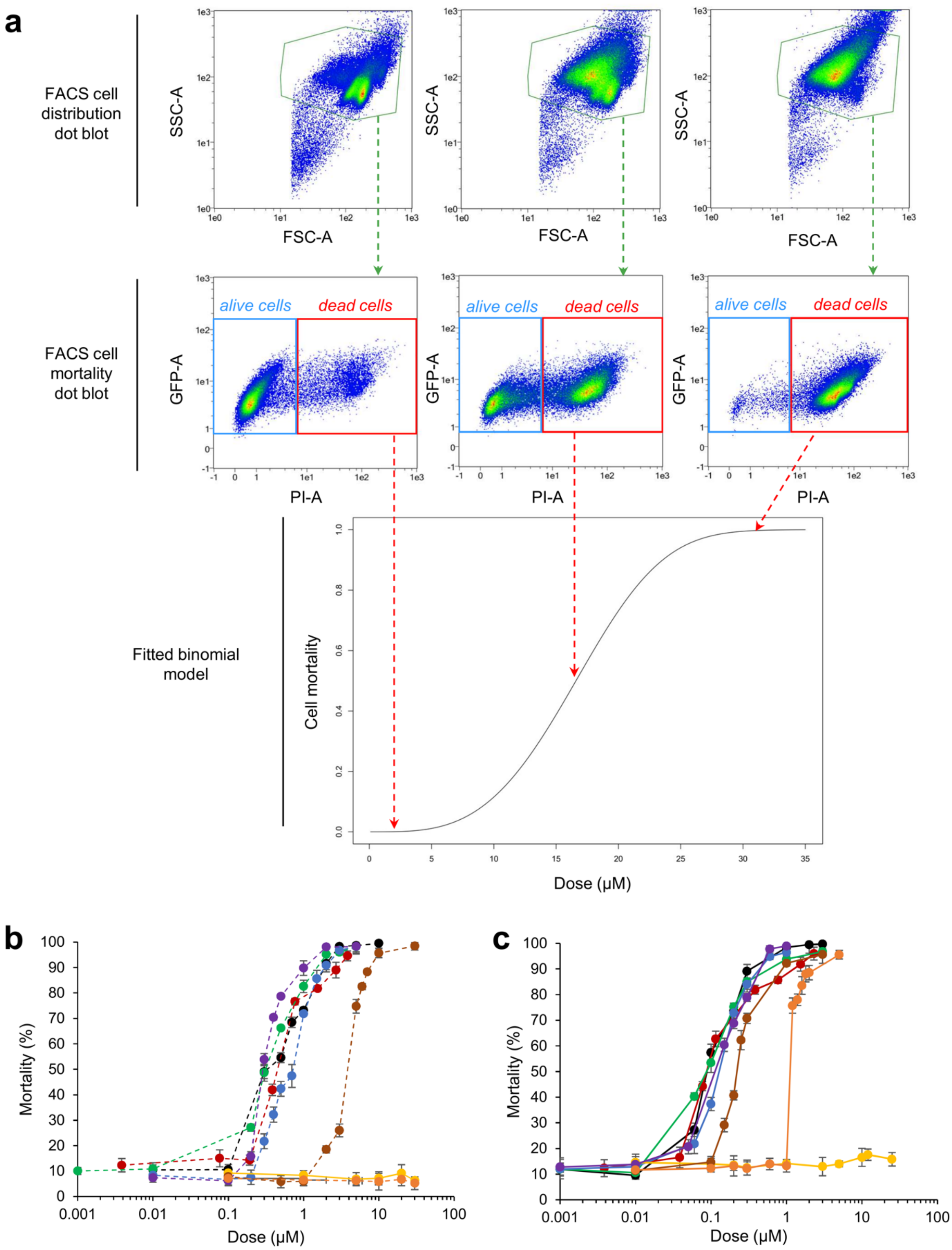

Supplementary Fig. 14. Cytotoxicity of WT and mutants Cyt1Aa protoxin and activated toxin monomers. a, Sigmoidal binomial model of mortality used to fit the FACS cell mortality data obtained by co-incubation with the toxin and propidium iodide (PI). Upon cell membrane rupture, PI penetrates cells and binds to their DNA, leading to an increase in its fluorescence at 617 nm, upon excitation at 475 nm. In the upper plots, only cells inside the green heptagon are considered for mortality analysis, which is conservative while allowing discarding most cell debris and cell clumps. The shape of the green zone was designed to encompass all

usable cells from all conditions and the same shape was used for all doses of all toxins. In the middle plots showing raw FACS cell mortality dot blot data, red squares indicate the cell population considered positive for PI insertion and therefore counted as dead, while the blue squares indicate living cells. **b-c**, Protoxin (**b**) and activated toxin monomers (**c**) of WT (black), C7S (green), C190V (red), Y171F (purple), D11N (blue), Q168E (yellow), E32Q (brown) and E45Q (orange) Cyt1Aa were assayed for toxicity against HEK293 cells at different concentrations. Toxicity of the E32Q (brown) mutant is significantly different from that of the WT in the protoxin form, but not in the activated toxin form. This result evidences that stabilization of the DS dimer results in inhibition of toxicity. Toxicity of the E45Q (orange) mutant is abrogated in the protoxin form and strongly affected in the activated toxin form, in line with the dual role of this residue in stabilizing the DS dimer and in tethering the αC/αD hairpin atop the β-sheet in each monomer, respectively. The Q168E (yellow) mutant shows no cytotoxic activity, in both the toxin and protoxin form, suggesting that this mutation effects on the interaction of the toxin with cell membranes. Values are indicated as mean ± SD. Source data are provided as a Source Data file.

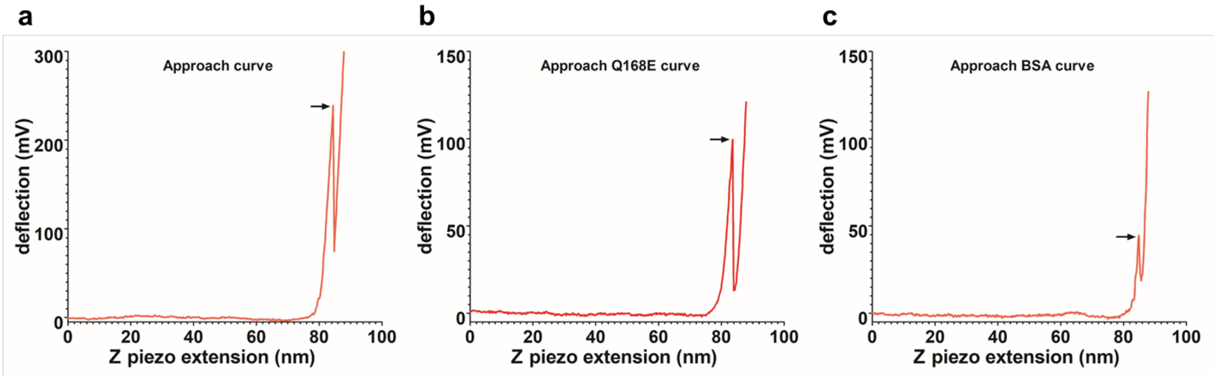

Supplementary Fig. 15. Force-distance curves confirms the presence of membrane bilayers in AFM experiments. Presence of the bilayer was confirmed before addition of WT Cyt1Aa in the kinetics experiments (**a**) and after the addition of Q168E mutant (**b**) and BSA control (**c**). Corresponding results are presented in Fig. 5d-k, 5l and 5m, respectively. Black arrows indicate the typical puncturing of the membrane.

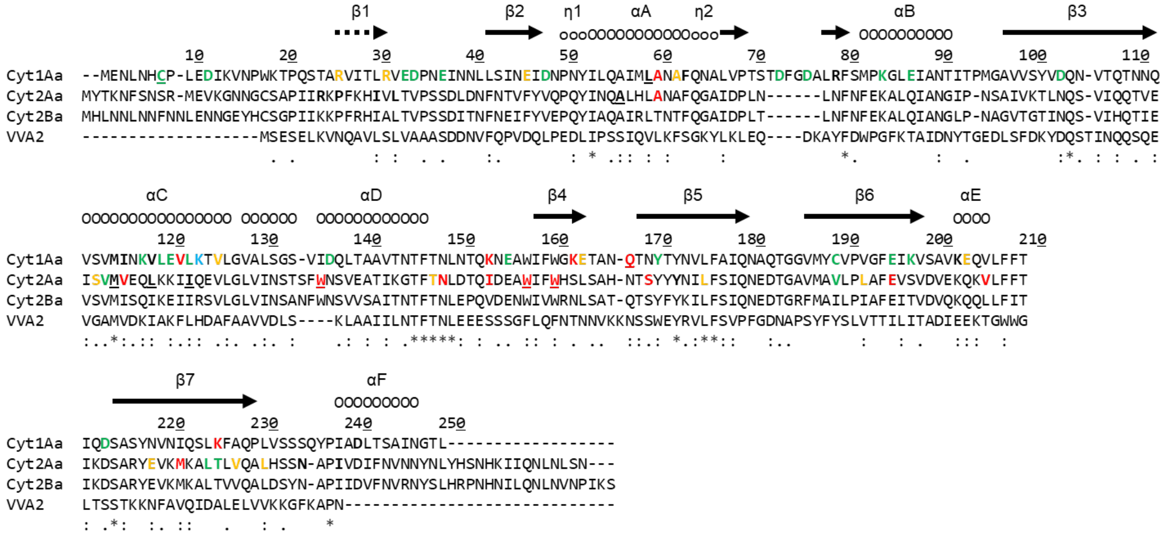

Supplementary Fig. 16. Sequence conservation across members and affiliates of the Cyt family of proteins, and known effects of point-mutations. Alignment of Cyt1Aa, Cyt2Aa, Cyt2Ba and VVA2 sequences was performed using the ClustalW software available in the PBIL Expasy tool (*52*) (http://npsa-pbil.ibcp.fr/cgi-bin/npsa_automat.pl?page=/NPSA/npsa_clustalw.html). The secondary structure of the Cyt1Aa protoxin, derived from DSSP calculation, is highlighted on top of the aligned sequences. Arrows represent β-sheets (numbered from β1 to β7, with β1 belonging to the N-terminal propeptide), "O" represent α-helices (from αA to αF) and "o" represent 3_{10} helices (from η1 to η3). β1 is represented by a dashed arrow. For each toxin, residues that have been challenged by point-mutations are indicated in bold. Residues whose mutation affected the solubility of the crystal are underlined. Residues whose mutation increased, decreased, suppressed or did not affect the toxicity are coloured in blue, orange, red and green, respectively. Residue conservation between the four sequences is indicated by '*' (identity), ':' (highly conserved) and '.' (conserved). Information regarding the effect of point mutations performed in the present study and by others before us are centralized in the Supplementary Table 3.

Supplementary Tables 1-4

Supplementary Table 1. Summary of crystal packing interactions.

Supplementary Table 2. Output and statistics from the GLM analyses used to calculate the pH solubilizing 50% of crystals (SP_{50}) and the dose killing 50% of cell population (LC_{50}).

Supplementary Table 3. Effect of point Cyt1Aa and Cyt2Aa mutations performed in the present study and by others before us on crystal formation, solubility and toxin toxicity.

Supplementary Table 4. List of all primers used for plasmid construction.

Supplementary Table 1 | Summary of crystal packing interactions.

Interface number	Description	
**Overall**	The asymmetric unit monomer is shown as a ribbon colored in slate. In the natural crystals, it establishes contact with nine symmetry-related mates, shown as ribbons of different colors, with each molecule having the same colour coding in all panels. In the following panels, atoms involved in the discussed interface(s) are shown as spheres, with carbon atoms colored as the ribbon, and nitrogen, oxygen and sulfur atoms colored in blue, red and yellow, respectively. Below, the reported H-bonds are those found by PISA, further filtered on the basis of a minimal distance of 3.2 Å between non-hydrogen atoms. We also used PISA to calculate the per-monomer buried surface area. For the sake of concision, only H-bonds expected to be pH-sensitive are explicitly discussed below. These include H-bonds wherein an acidic residue is involved, with specific focus on those wherein the acidic oxygen is an obligate donor, because such H-bonds are bound to break upon deprotonation due to elevation of pH.	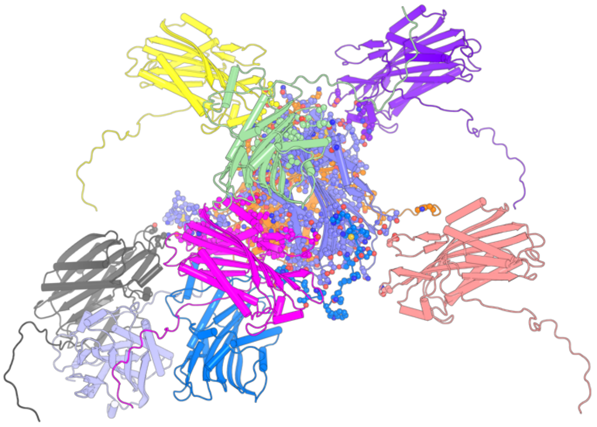

**1**	Domain swapped interface, classified as a stable block by PISA (predicted ∆G= -27.1 kcal/mol). Residues 6, 18-60, 71, 134, 137, 138, 140, 141, 144, 145, 149-160, 181+203, 208-213 and 233-247 (C-terminal propeptide) from both chains are involved. The N-terminal (β1 and β2) and C-terminal (αF) propeptides as well as the αC/αD hairpin are buried at this interface which features 46 H-bonds and two salt-bridges. The salt-bridges involve R30 (NH1) and D45 (OE2) from facing monomers in the DS dimer. H-bonds previewed to disrupt upon pH elevation include those between facing T23(OG1) and D33(OD1), and N181(ND2) and E32(OE2), respectively, but most importantly, that between E32(OE1) and E45 (OE2) wherein the latter atom is an obligate donor.<br><br>Per monomer buried surface area: 3077 Å^2	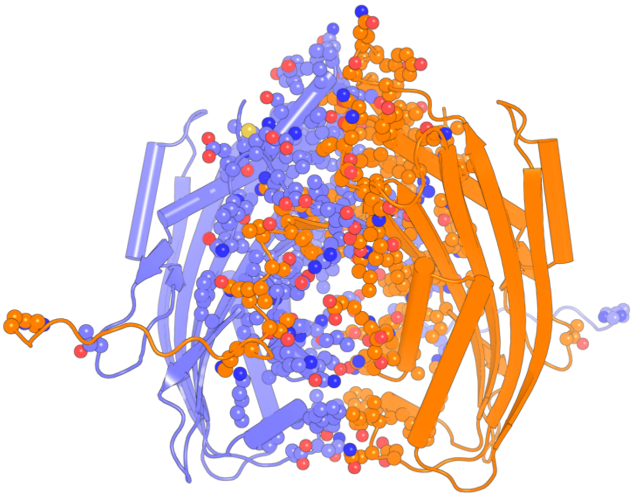
**2**	Residues 13-19 (N-terminal propeptide), 103, 106 (β3), 111, 113, 114, 117, 118, 121, 122, 124, 125, 129, 140 (αC/αD) and 212 (αE) from the asymmetric unit monomer interact with residues 33, 36, 37 (β1-β2 loop), 53, 54, 57, 63, 64, 66, 68, 71, 75, 77, 83, 86, 87, 90, 92, 93 (αA/αB), 228, 231 (β7), 233, 235, 236, 237, 240, 248 and 249 (C-terminal propeptide) from the symmetry-related mate.<br><br>Four H-bonds are found at this interface, of which one involves the side chain oxygen of an acidic residue, viz. that between N15(ND2) and D240(OD2). A salt-bridge is also found at this interface, viz. between K118(NZ) and D75(OD1).	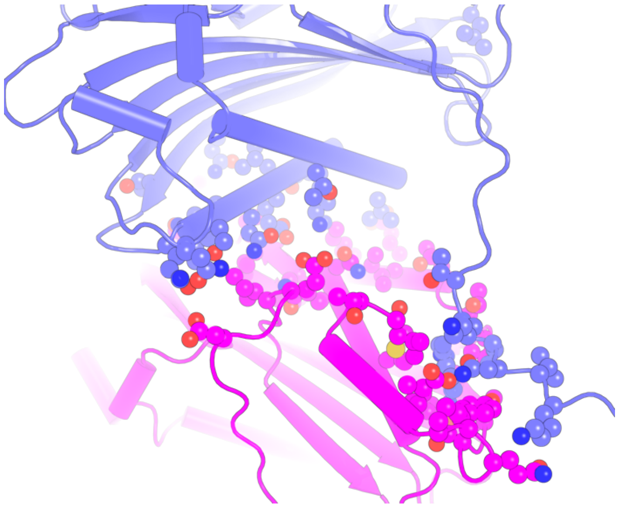

	Per monomer buried surface area: 770 Å^2	
**3**	Residues 102-104 (β3), 108-114 (β4), 167-170 (β4-β5 loop), 196-200 and 216-220 (αE) from the asymmetric unit monomer interact with residues 6-23 from the symmetry-related mate.<br><br>Six H-bonds are found at this interface, of which a single involves the side chain oxygen of an acidic residues, viz. that between Q168(NE2) and D11(OD1). A salt-bridge is also found, viz. between K198(NZ) and D11(OD1).<br><br>Per monomer buried surface area: 646 Å^2	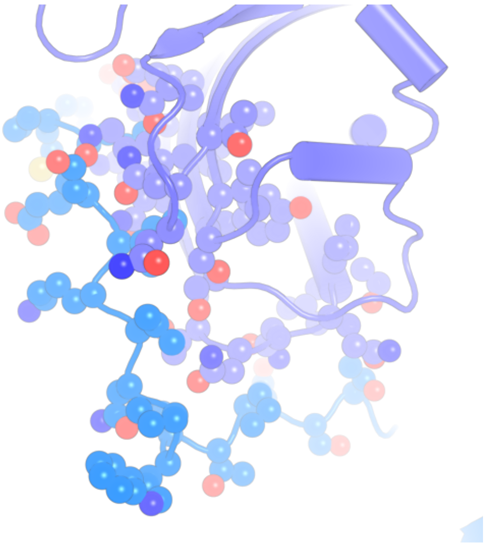
**4**	Residues 89, 94-98 (αB-β3 loop), 130 (αC/αD hairpin), 182-189 (β5-β6 loop), and 227-229 (β7), from both chains are involved.<br><br>There is no H-bond or salt-bridge at this interface.<br><br>Per monomer buried surface area: 411 Å^2	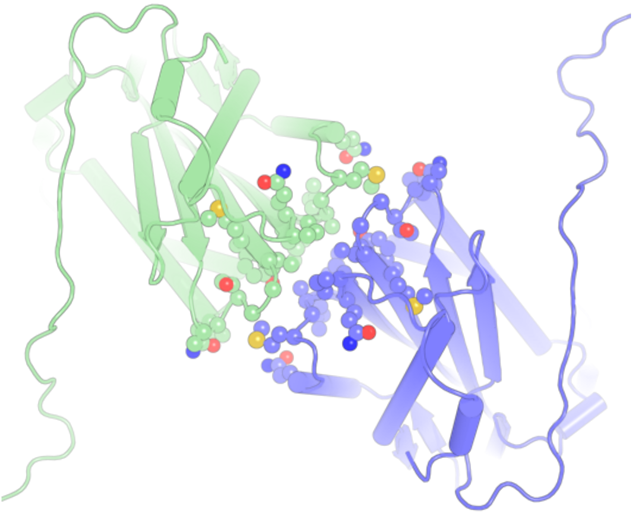

**5**	Residues 35, 36 (β1-β2 loop), 239, 242, 243, 246, 248, 249 (C-terminal propeptide) from the asymmetric unit monomer interact with residues 151, 154, 155, 164 (αD-β4 loop), 204, 207, 208 (αE) from the symmetry-related mate.<br><br>The three H-bonds found at this interface involve side chain oxygens of acidic residues, viz. E36(OE2), which interacts with N151(ND2); and E204(OE1) and (OE2), which interact with T248(OG) and N35(ND2), respectively.<br><br>Per monomer buried surface area: 225 Å^2	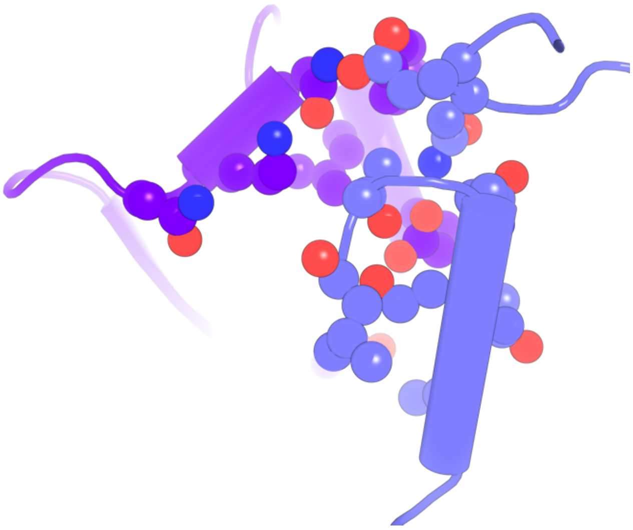
**6**	Residues 48-50 (β2-αA loop), 232, 234 and 235 (C-terminal loop) from the asymmetric unit monomer interact with residues 164-166 (β4-β5 loop) and 202, 204, 205 (αE) from the symmetry-related mate.<br><br>There is no H-bond or salt-bridge at this interface, which nonetheless participates in reducing access to Q168.<br><br>Per monomer buried surface area: 224 Å^2	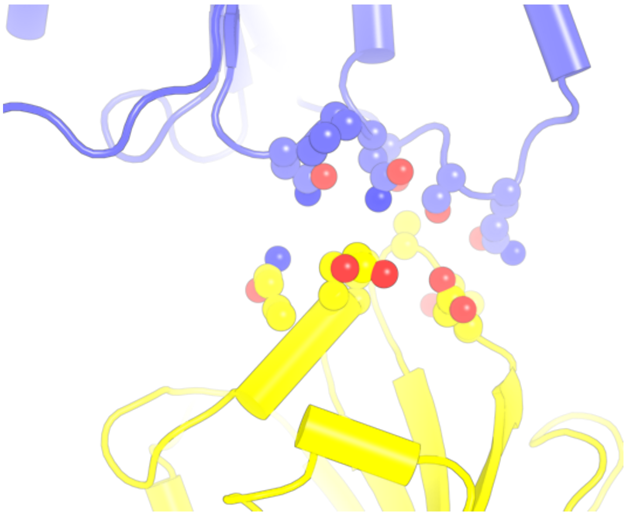

**7**	Disulfide-bridged interface.<br><br>Residues 6-8 (N-terminal propeptide) from both chains are involved, with a disulfide bridge at position C7.<br><br>There is no H-bond or salt-bridge at this interface.<br><br>Per monomer buried surface area: 71 Å^2	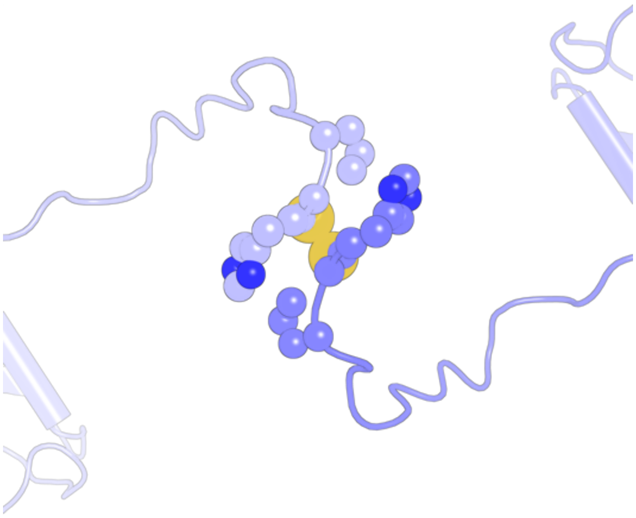
**8**	Residues 72 (tip of the αA/αB hairpin) and 167 (β4-β5 loop) from both chains are involved.<br><br>There is no H-bond or salt-bridge at this interface, which nonetheless participates in reducing access to Q168.<br><br>Per monomer buried surface area: 37 Å^2	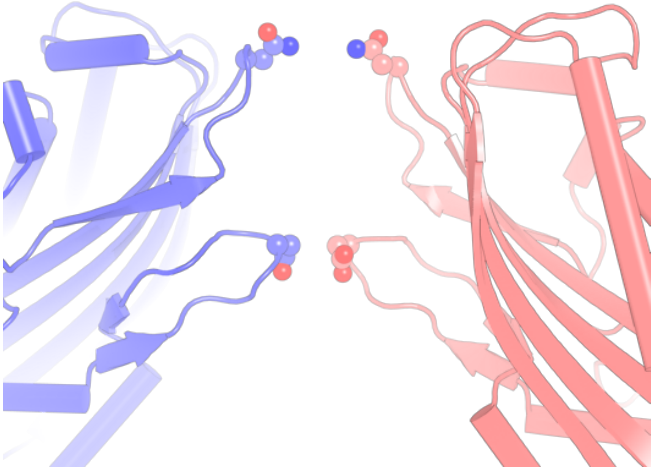
**9**	Residues 9 and 10 (N-terminal propeptide) from the asymmetric unit monomer interact with residues 71 (tip of the αA/αB hairpin) and 163 (β4-β5 loop) from the symmetry-related mate.<br><br>There is no H-bond or salt-bridge at this interface.<br><br>Per monomer buried surface area: 27 Å^2	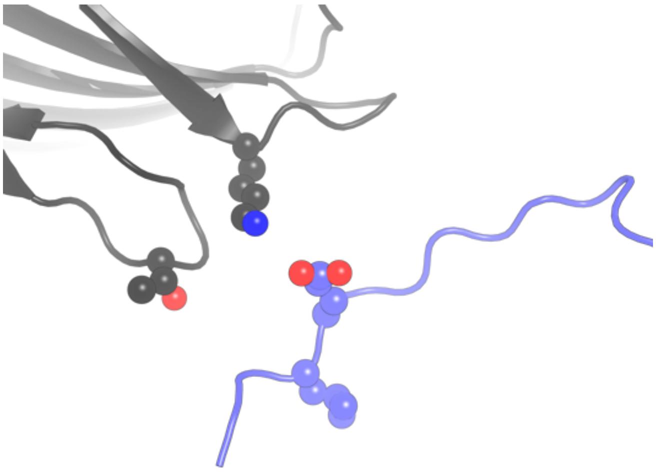

Supplementary Table 2. Output and statistics from the GLM analyses used to calculate the pH solubilizing 50% of crystals (SP_{50}) and the dose killing 50% of cell population (LC_{50}).

Model fitting & calculation of pH for solubilizing 50% of crystals

```
*****************
Cyt1Aa WT
*****************
Deviance results:
   probit   logit  cloglog   cauchit
1 3.740734 2.263416 2.007052 0.3755288

Call:
glm(formula = x ~ log(d), family = family(i), data = status)

Deviance Residuals:
    Min       1Q   Median       3Q      Max
-0.25531  -0.02203  -0.00035   0.01654   0.38683

Coefficients:
         Estimate Std. Error z value Pr(>|z|)
(Intercept)  -503.7     633.0  -0.796    0.426
log(d)       208.7     262.9   0.794    0.427

(Dispersion parameter for binomial family taken to be 1)

   Null deviance: 20.52227  on 26  degrees of freedom
Residual deviance:  0.37553  on 25  degrees of freedom
AIC: 6.2365

Number of Fisher Scoring iterations: 9


$ld.est
          LD    SE    LCL    UCL
p = 0.50: 11.175 1.013 10.901 11.456
p = 0.90: 11.341 1.028 10.743 11.973
p = 0.95: 11.518 1.047 10.519 12.613

$comparison
[1] 1

$R2_Naglekerke
[1] 0.983

*****************
C190V
*****************
Deviance results:
   probit   logit  cloglog   cauchit
1 4.236168 3.019759 2.195564 0.4530417

Call:
glm(formula = x ~ log(d), family = family(i), data = status)
```

```
Deviance Residuals:
    Min       1Q   Median       3Q      Max
-0.14563  -0.02570   0.04302   0.12708   0.34333

Coefficients:
            Estimate Std. Error z value Pr(>|z|)
(Intercept)  -206.73     169.38  -1.221    0.222
log(d)         85.99      70.47   1.220    0.222

(Dispersion parameter for binomial family taken to be 1)

    Null deviance: 18.18970  on 26  degrees of freedom
Residual deviance:  0.45304  on 25  degrees of freedom
AIC: 9.3694

Number of Fisher Scoring iterations: 8


$ld.est
            LD    SE    LCL    UCL
p = 0.50: 11.069 1.012 10.811 11.332
p = 0.90: 11.472 1.033 10.772 12.218
p = 0.95: 11.912 1.064 10.552 13.447

$comparison
[1] 1

$R2_Naglekerke
[1] 0.978

*****************
C7S
*****************
Deviance results:
    probit    logit  cloglog   cauchit
1 1.363429 1.059124 2.115507 0.4529237

Call:
glm(formula = x ~ log(d), family = family(i), data = status)

Deviance Residuals:
    Min       1Q   Median       3Q      Max
-0.16446  -0.04119   0.02593   0.12109   0.25711

Coefficients:
            Estimate Std. Error z value Pr(>|z|)
(Intercept)  -125.96      94.28  -1.336    0.182
log(d)         55.75      41.73   1.336    0.182

(Dispersion parameter for binomial family taken to be 1)

    Null deviance: 17.94163  on 26  degrees of freedom
```

```
Residual deviance:  0.45292  on 25  degrees of freedom
AIC: 9.8699

Number of Fisher Scoring iterations: 8


$ld.est
          LD    SE   LCL    UCL
p = 0.50:  9.578 1.019 9.234  9.935
p = 0.90: 10.122 1.047 9.254 11.070
p = 0.95: 10.727 1.091 9.042 12.726

$comparison
[1] 1

$R2_Naglekerke
[1] 0.977

*****************
D11N
*****************
Deviance results:
   probit   logit  cloglog  cauchit
1 6.076062 4.377727 3.466947 0.8981412

Call:
glm(formula = x ~ log(d), family = family(i), data = status)

Deviance Residuals:
    Min       1Q   Median       3Q      Max
-0.12277  -0.01163   0.06309   0.22727   0.37583

Coefficients:
         Estimate Std. Error z value Pr(>|z|)
(Intercept)  -299.6     268.6  -1.116    0.265
log(d)        125.6     112.5   1.116    0.264

(Dispersion parameter for binomial family taken to be 1)

   Null deviance: 23.30151  on 26  degrees of freedom
Residual deviance:  0.89814  on 25  degrees of freedom
AIC: 8.6484

Number of Fisher Scoring iterations: 10


$ld.est
          LD    SE    LCL    UCL
p = 0.50: 10.869 1.009 10.686 11.056
p = 0.90: 11.139 1.023 10.648 11.652
p = 0.95: 11.430 1.046 10.460 12.489
```

```
$comparison
[1] 1

$R2_Naglekerke
[1] 0.976

*****************
E32Q
*****************
Deviance results:
   probit   logit  cloglog  cauchit
1 4.434995 3.183111 2.331268 0.3711623

Call:
glm(formula = x ~ log(d), family = family(i), data = status)

Deviance Residuals:
    Min       1Q    Median       3Q      Max
-0.08973  -0.02631   0.07428   0.09440   0.39096

Coefficients:
            Estimate Std. Error z value Pr(>|z|)
(Intercept) -240.50     204.51  -1.176    0.240
log(d)        99.17      84.28   1.177    0.239

(Dispersion parameter for binomial family taken to be 1)

    Null deviance: 16.01616  on 26  degrees of freedom
Residual deviance:  0.37116  on 25  degrees of freedom
AIC: 7.707

Number of Fisher Scoring iterations: 8


$ld.est
             LD    SE    LCL    UCL
p = 0.50: 11.305 1.016 10.969 11.652
p = 0.90: 11.661 1.030 11.005 12.357
p = 0.95: 12.048 1.057 10.813 13.424

$comparison
[1] 1

$R2_Naglekerke
[1] 0.978

*****************
E45Q
*****************
Deviance results:
   probit   logit  cloglog  cauchit
1 4.823316 3.397681 2.446333 0.536153
```

```
Call:
glm(formula = x ~ log(d), family = family(i), data = status)

Deviance Residuals:
    Min       1Q   Median       3Q      Max
-0.07358  0.01090  0.07056  0.11252  0.40171

Coefficients:
         Estimate Std. Error z value Pr(>|z|)
(Intercept)  -267.5     264.2  -1.013    0.311
log(d)       110.0     108.6   1.013    0.311

(Dispersion parameter for binomial family taken to be 1)

    Null deviance: 15.90130  on 26  degrees of freedom
Residual deviance:  0.53615  on 25  degrees of freedom
AIC: 8.0536

Number of Fisher Scoring iterations: 9


$ld.est
          LD    SE    LCL    UCL
p = 0.50: 11.376 1.011 11.141 11.616
p = 0.90: 11.699 1.029 11.068 12.366
p = 0.95: 12.048 1.058 10.790 13.453

$comparison
[1] 1

$R2_Naglekerke
[1] 0.972

*****************
Q168E
*****************
Deviance results:
    probit    logit cloglog   cauchit
1 1.616793 1.142576 1.69814 0.5356339

Call:
glm(formula = x ~ log(d), family = family(i), data = status)

Deviance Residuals:
    Min       1Q   Median       3Q      Max
-0.15309  0.06354  0.08847  0.17467  0.24883

Coefficients:
         Estimate Std. Error z value Pr(>|z|)
(Intercept) -216.29    179.82  -1.203    0.229
log(d)       94.15     78.24   1.203    0.229
```

```
(Dispersion parameter for binomial family taken to be 1)

    Null deviance: 23.95575  on 26  degrees of freedom
Residual deviance:  0.53563  on 25  degrees of freedom
AIC: 8.7116

Number of Fisher Scoring iterations: 9


$ld.est
          LD    SE   LCL    UCL
p = 0.50:  9.947 1.012 9.715 10.185
p = 0.90: 10.278 1.029 9.711 10.877
p = 0.95: 10.637 1.058 9.528 11.875

$comparison
[1] 1

$R2_Naglekerke
[1] 0.983

*****************
Y171F
*****************
Deviance results:
   probit    logit  cloglog  cauchit
1 4.488139 3.640097 2.401891 1.663891

Call:
glm(formula = x ~ log(d), family = family(i), data = status)

Deviance Residuals:
    Min       1Q   Median       3Q      Max
-0.33055   0.05185   0.13613   0.26819   0.51428

Coefficients:
            Estimate Std. Error z value Pr(>|z|)
(Intercept)  -128.02      92.85  -1.379    0.168
log(d)         53.59      38.90   1.378    0.168

(Dispersion parameter for binomial family taken to be 1)

    Null deviance: 18.3382  on 26  degrees of freedom
Residual deviance:  1.6639  on 25  degrees of freedom
AIC: 12.33

Number of Fisher Scoring iterations: 10


$ld.est
          LD    SE    LCL    UCL
```

```
p = 0.50: 10.902 1.015 10.590 11.223
p = 0.90: 11.547 1.047 10.556 12.630
p = 0.95: 12.266 1.092 10.314 14.586

$comparison
[1] 1

$R2_Naglekerke
[1] 0.936

*****************
Cyt1Aa WT + DTT
*****************
Deviance results:
   probit    logit  cloglog   cauchit
1 2.389225 2.008871 3.969117 0.8496498

Call:
glm(formula = x ~ log(d), family = family(i), data = status)

Deviance Residuals:
    Min       1Q    Median       3Q      Max
-0.33320  -0.16867  -0.12742  -0.02102   0.27780

Coefficients:
            Estimate Std. Error z value Pr(>|z|)
(Intercept)  -147.07     114.21  -1.288    0.198
log(d)         64.51      50.05   1.289    0.197

(Dispersion parameter for binomial family taken to be 1)

    Null deviance: 20.70769  on 25  degrees of freedom
Residual deviance:  0.84965  on 24  degrees of freedom
AIC: 9.9208

Number of Fisher Scoring iterations: 8


$ld.est
           LD    SE   LCL    UCL
p = 0.50:  9.776 1.016 9.484 10.076
p = 0.90: 10.253 1.040 9.500 11.066
p = 0.95: 10.781 1.079 9.285 12.518

$comparison
[1] 1

$R2_Naglekerke
[1] 0.961

*****************
C190V + DTT
```

```
*****************

   probit   logit  cloglog   cauchit
1 2.254215 1.608174 3.996601 0.5829255

Call:
glm(formula = x ~ log(d), family = family(i), data = status)

Deviance Residuals:
    Min       1Q   Median       3Q      Max
-0.35542  -0.04057   0.04221   0.10167   0.22353

Coefficients:
         Estimate Std. Error z value Pr(>|z|)
(Intercept)  -226.78    220.58  -1.028    0.304
log(d)        99.78     96.91   1.030    0.303

(Dispersion parameter for binomial family taken to be 1)

   Null deviance: 23.12126  on 25  degrees of freedom
Residual deviance:  0.58293  on 24  degrees of freedom
AIC: 8.5075

Number of Fisher Scoring iterations: 8


$ld.est
          LD    SE   LCL    UCL
p = 0.50:  9.706 1.012 9.487  9.930
p = 0.90: 10.010 1.029 9.458 10.594
p = 0.95: 10.340 1.061 9.206 11.613

$comparison
[1] 1

$R2_Naglekerke
[1] 0.977

*****************
C7S + DTT
*****************

   probit   logit  cloglog   cauchit
1 2.469138 1.755875 4.308441 0.2826912

Call:
glm(formula = x ~ log(d), family = family(i), data = status)

Deviance Residuals:
     Min        1Q    Median        3Q       Max
-0.221603  -0.056826  -0.001174   0.054000   0.225291
```

```
Coefficients:
        Estimate Std. Error z value Pr(>|z|)
(Intercept) -208.33    191.35  -1.089    0.276
log(d)        91.98     84.16   1.093    0.274

(Dispersion parameter for binomial family taken to be 1)

   Null deviance: 22.25151  on 25  degrees of freedom
Residual deviance:  0.28269  on 24  degrees of freedom
AIC: 7.6208

Number of Fisher Scoring iterations: 8


$ld.est
          LD    SE   LCL    UCL
p = 0.50:  9.631 1.020 9.270 10.007
p = 0.90:  9.959 1.029 9.408 10.542
p = 0.95: 10.315 1.060 9.209 11.555

$comparison
[1] 1

$R2_Naglekerke
[1] 0.989

*****************
Y171F + DTT
*****************

    probit    logit  cloglog  cauchit
1 2.828521 2.692725 5.749051 2.393754

Call:
glm(formula = x ~ log(d), family = family(i), data = status)

Deviance Residuals:
    Min       1Q   Median       3Q      Max
-0.37737  -0.27014  -0.19502  -0.08495   0.30468

Coefficients:
        Estimate Std. Error z value Pr(>|z|)
(Intercept)  -99.88     51.40  -1.943   0.0520 .
log(d)        44.36     22.82   1.944   0.0519 .
---
Signif. codes:  0 ‘***’ 0.001 ‘**’ 0.01 ‘*’ 0.05 ‘.’ 0.1 ‘ ’ 1

(Dispersion parameter for binomial family taken to be 1)

   Null deviance: 37.6225  on 47  degrees of freedom
Residual deviance:  2.3938  on 46  degrees of freedom
AIC: 16.584
```

```
Number of Fisher Scoring iterations: 8


$ld.est
         LD   SE  LCL   UCL
p = 0.50:  9.503 1.016 9.215  9.800
p = 0.90: 10.186 1.039 9.443 10.987
p = 0.95: 10.956 1.077 9.468 12.679

$comparison
[1] 1

$R2_Naglekerke
[1] 0.962

*****************
D11N + DTT
*****************

   probit   logit  cloglog  cauchit
1 4.072643 3.144755 7.950213 1.297011

Call:
glm(formula = x ~ log(d), family = family(i), data = status)

Deviance Residuals:
    Min       1Q   Median       3Q      Max
-0.35280  -0.19036  -0.09556   0.02622   0.23885

Coefficients:
         Estimate Std. Error z value Pr(>|z|)
(Intercept)  -148.19     82.41  -1.798   0.0721 .
log(d)       66.71     37.05   1.800   0.0718 .
---
Signif. codes:  0 '***' 0.001 '**' 0.01 '*' 0.05 '.' 0.1 ' ' 1

(Dispersion parameter for binomial family taken to be 1)

    Null deviance: 42.682  on 47  degrees of freedom
Residual deviance:  1.297  on 46  degrees of freedom
AIC: 12.416

Number of Fisher Scoring iterations: 8


$ld.est
         LD   SE  LCL   UCL
p = 0.50:  9.220 1.016 8.935  9.514
p = 0.90:  9.655 1.029 9.122 10.220
p = 0.95: 10.135 1.055 9.124 11.258
```

$comparison
[1] 1

$R2_Naglekerke
[1] 0.976

****
Q168E + DTT
****

```
    probit    logit  cloglog  cauchit
1 4.190581 3.609607 6.948139 1.897533

Call:
glm(formula = x ~ log(d), family = family(i), data = status)

Deviance Residuals:
     Min        1Q    Median        3Q       Max
-0.43735  -0.14579  -0.06320   0.05378   0.33611

Coefficients:
            Estimate Std. Error z value Pr(>|z|)
(Intercept)  -47.900     19.703  -2.431   0.0151 *
log(d)        22.437      9.193   2.441   0.0147 *
---
Signif. codes:  0 '***' 0.001 '**' 0.01 '*' 0.05 '.' 0.1 ' ' 1

(Dispersion parameter for binomial family taken to be 1)

    Null deviance: 37.3550  on 68  degrees of freedom
Residual deviance:  1.8975  on 67  degrees of freedom
AIC: 31.631

Number of Fisher Scoring iterations: 7


$ld.est
              LD    SE   LCL    UCL
p = 0.50:  8.456 1.019 8.152  8.771
p = 0.90:  9.699 1.058 8.690 10.825
p = 0.95: 11.204 1.120 8.968 13.997

$comparison
[1] 1

$R2_Naglekerke
[1] 0.955
```

****
E32Q + DTT
****
probit logit cloglog cauchit

```
1 1.299407 1.132439 2.620451 0.9753236

Call:
glm(formula = x ~ log(d), family = family(i), data = status)

Deviance Residuals:
    Min       1Q   Median       3Q      Max
-0.34378  -0.14796  -0.07592   0.01604   0.33836

Coefficients:
         Estimate Std. Error z value Pr(>|z|)
(Intercept)  -108.75     61.56  -1.767   0.0773 .
log(d)        47.64     26.98   1.766   0.0774 .
---
Signif. codes:  0 '***' 0.001 '**' 0.01 '*' 0.05 '.' 0.1 ' ' 1

(Dispersion parameter for binomial family taken to be 1)

    Null deviance: 29.03215  on 38  degrees of freedom
Residual deviance:  0.97532  on 37  degrees of freedom
AIC: 15.607

Number of Fisher Scoring iterations: 7


$ld.est
          LD    SE   LCL    UCL
p = 0.50:  9.804 1.015 9.518 10.098
p = 0.90: 10.458 1.041 9.665 11.316
p = 0.95: 11.193 1.080 9.620 13.023

$comparison
[1] 1

$R2_Naglekerke
[1] 0.969

*****************
E45Q + DTT
*****************
    probit     logit cloglog   cauchit
1 1.541617 0.9816774 2.19867 0.5073008

Call:
glm(formula = x ~ log(d), family = family(i), data = status)

Deviance Residuals:
    Min       1Q   Median       3Q      Max
-0.17223  -0.08874  -0.03659   0.06801   0.32434

Coefficients:
         Estimate Std. Error z value Pr(>|z|)
```

```
(Intercept) -181.17    111.29  -1.628    0.104
log(d)        78.88     48.44   1.628    0.103

(Dispersion parameter for binomial family taken to be 1)

    Null deviance: 35.4507  on 38  degrees of freedom
Residual deviance:  0.5073  on 37  degrees of freedom
AIC: 10.66

Number of Fisher Scoring iterations: 8


$ld.est
           LD    SE   LCL    UCL
p = 0.50:  9.943 1.016 9.644 10.250
p = 0.90: 10.338 1.029 9.779 10.930
p = 0.95: 10.771 1.053 9.740 11.912

$comparison
[1] 1

$R2_Naglekerke
[1] 0.989
```

Model fitting & calculation of concentration for killing 50% of HEK293 cell population

```
****************************
Cyt1Aa WT Solubilized
****************************

    probit    logit   cloglog   cauchit
1 0.3794631 0.4340959 0.1848265 0.8864705

$Resum

Call:
glm(formula = x ~ log(d), family = family(i), data = status)

Deviance Residuals:
     Min        1Q    Median        3Q       Max
-0.277588  -0.009484  -0.000094   0.077108   0.238158

Coefficients:
         Estimate Std. Error z value Pr(>|z|)
(Intercept)  -33.585    26.135  -1.285    0.199
log(d)         3.419     2.641   1.294    0.196

(Dispersion parameter for binomial family taken to be 1)

   Null deviance: 10.09516
Residual deviance:  0.18483
AIC: 5.9924

Number of Fisher Scoring iterations: 11


$ld.est
           LD   SE     LCL     UCL
p = 0.50: 16589.23 1.293 10017.98 27470.86
p = 0.90: 23568.57 1.294 14213.03 39082.27
p = 0.95: 25454.37 1.342 14310.56 45276.02

$comparison
[1] 0.9999972

$R2_Naglekerke
[1] 0.982

****************************
Cyt1Aa WT Solubilized DTT
****************************

    probit    logit   cloglog   cauchit
1 0.1649665 0.2317902 0.1500927 0.7561266

$Resum
```

```
Call:
glm(formula = x ~ log(d), family = family(i), data = status)

Deviance Residuals:
    Min        1Q    Median        3Q       Max
-0.181304  -0.078387   0.001252   0.032495   0.244431

Coefficients:
        Estimate Std. Error z value Pr(>|z|)
(Intercept)  -6.9367     5.2566  -1.320    0.187
log(d)        1.1126     0.8149   1.365    0.172

(Dispersion parameter for binomial family taken to be 1)

   Null deviance: 6.96673  on 9  degrees of freedom
Residual deviance: 0.15009  on 8  degrees of freedom
AIC: 7.8362

Number of Fisher Scoring iterations: 8


$ld.est
           LD    SE    LCL     UCL
p = 0.50:  366.939 1.938 100.322 1342.117
p = 0.90: 1079.481 2.002 277.056 4205.924
p = 0.95: 1367.525 2.251 278.915 6705.005

$comparison
[1] 0.9999988

$R2_Naglekerke
[1] 0.978


***************************
Cyt1Aa WT Activated
***************************

    probit    logit   cloglog   cauchit
1 0.0965895 0.122949 0.1449628 0.5113992

$Resum

Call:
glm(formula = x ~ log(d), family = family(i), data = status)

Deviance Residuals:
    Min       1Q   Median       3Q      Max
-0.20404  -0.01291   0.00056   0.02002   0.20730

Coefficients:
```

```
        Estimate Std. Error z value Pr(>|z|)
(Intercept)  -6.365     6.037  -1.054   0.292
log(d)       1.434     1.281   1.119   0.263

(Dispersion parameter for binomial family taken to be 1)

   Null deviance: 8.228597  on 8  degrees of freedom
Residual deviance: 0.096589  on 7  degrees of freedom
AIC: 6.0894

Number of Fisher Scoring iterations: 9


$ld.est
          LD   SE   LCL     UCL
p = 0.50:  84.690 1.763 27.866  257.386
p = 0.90: 207.019 2.200 44.154  970.633
p = 0.95: 266.719 2.635 39.943 1781.020

$comparison
[1] 0.999998

$R2_Naglekerke
[1] 0.988

****************************
Cyt1Aa WT Activated DTT
****************************

     probit     logit   cloglog  cauchit
1 0.001046012 0.01061426 0.03373632 0.2565856

$Resum

Call:
glm(formula = x ~ log(d), family = family(i), data = status)

Deviance Residuals:
      30        31        32        33        34        35        36
-0.0000004  -0.0121812   0.0071729  -0.0138087   0.0058974   0.0249088   0.0005185

Coefficients:
        Estimate Std. Error z value Pr(>|z|)
(Intercept)  -7.402     9.478  -0.781   0.435
log(d)       1.567     1.866   0.840   0.401

(Dispersion parameter for binomial family taken to be 1)

   Null deviance: 6.642721  on 6  degrees of freedom
Residual deviance: 0.001046  on 5  degrees of freedom
AIC: 5.6474
```

```
Number of Fisher Scoring iterations: 9


$ld.est
          LD   SE   LCL     UCL
p = 0.50: 112.575 1.966 29.925  423.491
p = 0.90: 255.048 2.213 53.760 1209.999
p = 0.95: 321.596 2.753 44.187 2340.603

$comparison
[1] 1

$R2_Naglekerke
[1] 1

***************************
Cyt1Aa C7S Solubilized
***************************


    probit    logit  cloglog  cauchit
1 0.06107853 0.1050184 0.0156711 0.5714266

$Resum

Call:
glm(formula = x ~ log(d), family = family(i), data = status)

Deviance Residuals:
    Min       1Q    Median       3Q      Max
-0.079987  -0.026321   0.000417   0.024336   0.063507

Coefficients:
        Estimate Std. Error z value Pr(>|z|)
(Intercept)  -9.154     8.913  -1.027   0.304
log(d)       1.481     1.405   1.054   0.292

(Dispersion parameter for binomial family taken to be 1)

   Null deviance: 7.870847  on 7  degrees of freedom
Residual deviance: 0.015671  on 6  degrees of freedom
AIC: 5.5209

Number of Fisher Scoring iterations: 9


$ld.est
           LD   SE    LCL     UCL
p = 0.50:  378.189 1.987  98.476 1452.414
p = 0.90:  850.900 2.041 210.230 3443.983
p = 0.95: 1016.419 2.274 203.059 5087.733

$comparison
```

[1] 0.9999999

$R2_Naglekerke
[1] 0.998

****
Cyt1Aa C7S Solubilized DTT
****

```
    probit     logit   cloglog   cauchit
1 0.02883597 0.05511702 0.0544168 0.4463203
```

$Resum

Call:
glm(formula = x ~ log(d), family = family(i), data = status)

Deviance Residuals:

```
     Min        1Q    Median        3Q       Max
-0.076026  -0.021628  -0.000001   0.055144   0.120252
```

Coefficients:

```
            Estimate Std. Error z value Pr(>|z|)
(Intercept)   -7.243      6.720  -1.078    0.281
log(d)         1.275      1.126   1.132    0.257
```

(Dispersion parameter for binomial family taken to be 1)

Null deviance: 7.723538 on 8 degrees of freedom
Residual deviance: 0.028836 on 7 degrees of freedom
AIC: 6.8016

Number of Fisher Scoring iterations: 9

$ld.est

```
               LD    SE     LCL      UCL
p = 0.50:  293.378 1.802  92.486  930.631
p = 0.90:  801.714 2.355 149.627 4295.657
p = 0.95: 1066.074 2.895 132.746 8561.542
```

$comparison
[1] 1

$R2_Naglekerke
[1] 0.996

****
Cyt1Aa C7S Activated
****

```
    probit     logit  cloglog  cauchit
1 0.04616299 0.08250545 0.1477006 0.4889797

$Resum

Call:
glm(formula = x ~ log(d), family = family(i), data = status)

Deviance Residuals:
    Min        1Q    Median        3Q       Max
-0.136788  -0.057998   0.001975   0.034705   0.122914

Coefficients:
        Estimate Std. Error z value Pr(>|z|)
(Intercept)  -5.016     4.526  -1.108    0.268
log(d)        1.157     0.959   1.206    0.228

(Dispersion parameter for binomial family taken to be 1)

   Null deviance: 6.734446  on 7  degrees of freedom
Residual deviance: 0.046163  on 6  degrees of freedom
AIC: 6.3614

Number of Fisher Scoring iterations: 9


$ld.est
          LD    SE    LCL     UCL
p = 0.50:  76.455 1.961 20.432  286.083
p = 0.90: 231.499 2.451 39.959 1341.165
p = 0.95: 316.918 3.004 36.712 2735.812

$comparison
[1] 0.999998

$R2_Naglekerke
[1] 0.993

***************************
Cyt1Aa C7S Activated DTT
***************************

     probit     logit  cloglog  cauchit
1 0.008946602 0.01529912 0.117163 0.1343936

$Resum

Call:
glm(formula = x ~ log(d), family = family(i), data = status)

Deviance Residuals:
```

```
      94        95        96        97        98        99       100 
-0.000046  -0.069995   0.028343   0.011586  -0.052025   0.020068   0.000658 

Coefficients:
            Estimate Std. Error z value Pr(>|z|)
(Intercept)   -5.990      8.044  -0.745    0.456
log(d)         1.379      1.623   0.850    0.395

(Dispersion parameter for binomial family taken to be 1)

    Null deviance: 6.8664476  on 6  degrees of freedom
Residual deviance: 0.0089466  on 5  degrees of freedom
AIC: 5.1517

Number of Fisher Scoring iterations: 10


$ld.est
             LD    SE    LCL      UCL
p = 0.50:  76.908 2.628 11.579  510.821
p = 0.90: 194.745 2.221 40.750  930.698
p = 0.95: 253.426 2.702 36.130 1777.605

$comparison
[1] 0.9999996

$R2_Naglekerke
[1] 0.999

****************************
Cyt1Aa C190V Solubilized
****************************

    probit     logit   cloglog   cauchit
1 0.515224 0.5857071 0.4154309 0.9587459

$Resum

Call:
glm(formula = x ~ log(d), family = family(i), data = status)

Deviance Residuals:
     Min        1Q    Median        3Q       Max  
-0.38446  -0.12604   0.02591   0.22413   0.25695  

Coefficients:
            Estimate Std. Error z value Pr(>|z|)
(Intercept)  -17.683     10.840  -1.631    0.103
log(d)         1.827      1.118   1.634    0.102

(Dispersion parameter for binomial family taken to be 1)
```

```
   Null deviance: 5.99511  on 7  degrees of freedom
Residual deviance: 0.41543  on 6  degrees of freedom
AIC: 6.6854

Number of Fisher Scoring iterations: 8


$ld.est
           LD   SE      LCL     UCL
p = 0.50: 13099.36 1.489  6005.352 28573.36
p = 0.90: 25275.32 1.603 10026.112 63717.82
p = 0.95: 29192.13 1.697 10350.440 82332.80

$comparison
[1] 0.9987208

$R2_Naglekerke
[1] 0.931

****************************
Cyt1Aa C190V Solubilized DTT
****************************

    probit    logit   cloglog  cauchit
1 0.3162798 0.2958012 0.6600754 0.403564

$Resum

Call:
glm(formula = x ~ log(d), family = family(i), data = status)

Deviance Residuals:
    Min       1Q   Median       3Q      Max
-0.38065  -0.14868  -0.00604   0.06943   0.20162

Coefficients:
         Estimate Std. Error z value Pr(>|z|)
(Intercept)  -18.169     14.801  -1.228    0.220
log(d)         2.963      2.391   1.239    0.215

(Dispersion parameter for binomial family taken to be 1)

   Null deviance: 8.1874  on 7  degrees of freedom
Residual deviance: 0.2958  on 6  degrees of freedom
AIC: 5.5428

Number of Fisher Scoring iterations: 8


$ld.est
           LD   SE    LCL     UCL
p = 0.50:  460.722 1.625 177.837 1193.591
```

```
p = 0.90:  967.235 2.125 220.847 4236.152
p = 0.95: 1244.706 2.506 205.608 7535.186

$comparison
[1] 0.9995172

$R2_Naglekerke
[1] 0.964

***************************
Cyt1Aa C190V Activated
***************************

    probit    logit   cloglog   cauchit
1 0.4967895 0.4434799 0.7994513 0.4662616

$Resum

Call:
glm(formula = x ~ log(d), family = family(i), data = status)

Deviance Residuals:
    Min       1Q   Median       3Q      Max
-0.51831  -0.18746   0.00001   0.09990   0.20761

Coefficients:
         Estimate Std. Error z value Pr(>|z|)
(Intercept)   -9.840      8.687  -1.133    0.257
log(d)        2.165      1.897   1.141    0.254

(Dispersion parameter for binomial family taken to be 1)

   Null deviance: 6.97173  on 7  degrees of freedom
Residual deviance: 0.44348  on 6  degrees of freedom
AIC: 6.3819

Number of Fisher Scoring iterations: 7


$ld.est
           LD    SE    LCL     UCL
p = 0.50:  94.225 1.778 30.500  291.088
p = 0.90: 260.010 2.915 31.940 2116.634
p = 0.95: 367.201 3.799 26.833 5024.940

$comparison
[1] 0.9984599

$R2_Naglekerke
[1] 0.936

***************************
```

Cyt1Aa C190V Activated DTT
****

```
   probit    logit   cloglog  cauchit
1 0.307109 0.3047502 0.5363065 0.4984556
```

$Resum

Call:
glm(formula = x ~ log(d), family = family(i), data = status)

Deviance Residuals:

```
    Min       1Q   Median       3Q      Max
-0.44611  -0.14211   0.00938   0.10477   0.16973
```

Coefficients:

```
        Estimate Std. Error z value Pr(>|z|)
(Intercept)  -9.913     8.040  -1.233    0.218
log(d)        2.098     1.704   1.231    0.218
```

(Dispersion parameter for binomial family taken to be 1)

   Null deviance: 7.02340 on 7 degrees of freedom
Residual deviance: 0.30475 on 6 degrees of freedom
AIC: 6.4747

Number of Fisher Scoring iterations: 7

$ld.est

```
           LD    SE    LCL      UCL
p = 0.50: 112.815 1.820 34.900  364.677
p = 0.90: 321.555 2.951 38.548 2682.331
p = 0.95: 459.145 3.794 33.654 6264.109
```

$comparison
[1] 0.9994738

$R2_Naglekerke
[1] 0.957

****
Cyt1Aa D11N Activated
****

```
     probit     logit    cloglog  cauchit
1 0.03329603 0.06486176 0.03974787 0.3964164
```

$Resum

Call:
glm(formula = x ~ log(d), family = family(i), data = status)

```
Deviance Residuals:
    Min       1Q   Median       3Q      Max
-0.100045  -0.022087  0.007431  0.048828  0.108289

Coefficients:
        Estimate Std. Error z value Pr(>|z|)
(Intercept)  -6.777     5.543  -1.223   0.221
log(d)       1.454     1.135  1.280   0.200

(Dispersion parameter for binomial family taken to be 1)

   Null deviance: 7.112716  on 7  degrees of freedom
Residual deviance: 0.033296  on 6  degrees of freedom
AIC: 6.2545

Number of Fisher Scoring iterations: 8


$ld.est
          LD   SE   LCL     UCL
p = 0.50: 105.890 1.675 38.514  291.134
p = 0.90: 255.711 2.098 59.847 1092.576
p = 0.95: 328.317 2.448 56.773 1898.640

$comparison
[1] 0.9999992

$R2_Naglekerke
[1] 0.995

****************************
Cyt1Aa Y171F Activated
****************************
    probit    logit   cloglog  cauchit
1 0.06029828 0.1100342 0.08884954 0.4867741

$Resum

Call:
glm(formula = x ~ log(d), family = family(i), data = status)

Deviance Residuals:
    Min       1Q   Median       3Q      Max
-0.075906  -0.062219  -0.007244  0.075850  0.183879

Coefficients:
        Estimate Std. Error z value Pr(>|z|)
(Intercept) -5.5100    4.4370  -1.242   0.214
log(d)      1.1937    0.8775  1.360   0.174

(Dispersion parameter for binomial family taken to be 1)
```

```
   Null deviance: 6.780142  on 7  degrees of freedom
Residual deviance: 0.060298  on 6  degrees of freedom
AIC: 5.9308

Number of Fisher Scoring iterations: 8


$ld.est
          LD   SE   LCL     UCL
p = 0.50: 101.097 1.906 28.571  357.724
p = 0.90: 295.812 2.170 64.800 1350.375
p = 0.95: 401.050 2.554 63.838 2519.501

$comparison
[1] 0.9999955

$R2_Naglekerke
[1] 0.991

****************************
Cyt1Aa E32Q Activated
****************************

    probit   logit  cloglog  cauchit
1 0.1461402 0.177348 0.2361443 0.4487348

$Resum

Call:
glm(formula = x ~ log(d), family = family(i), data = status)

Deviance Residuals:
     117       118       119       120       121       122       123
-0.000001  -0.295560   0.223260  -0.045998   0.023806  -0.078205   0.011880

Coefficients:
        Estimate Std. Error z value Pr(>|z|)
(Intercept)  -12.772     10.679  -1.196    0.232
log(d)        2.400      2.007   1.196    0.232

(Dispersion parameter for binomial family taken to be 1)

   Null deviance: 4.77429  on 6  degrees of freedom
Residual deviance: 0.14614  on 5  degrees of freedom
AIC: 7.0578

Number of Fisher Scoring iterations: 8


$ld.est
          LD   SE    LCL     UCL
```

```
p = 0.50: 204.746 1.308 120.943  346.619
p = 0.90: 349.245 1.696 124.015  983.530
p = 0.95: 406.327 1.897 115.788 1425.890

$comparison
[1] 0.9995878

$R2_Naglekerke
[1] 0.969


***************************
Cyt1Aa E45Q Activated
***************************

    probit    logit  cloglog   cauchit
1 0.8462281 0.7179605 1.263394 0.3647874

$Resum

Call:
glm(formula = x ~ log(d), family = family(i), data = status)

Deviance Residuals:
    Min       1Q    Median       3Q       Max
-0.35180  -0.17385  -0.08859   0.01740   0.10510

Coefficients:
         Estimate Std. Error z value Pr(>|z|)
(Intercept) -278.46    628.07  -0.443    0.658
log(d)       39.48     88.60   0.446    0.656

(Dispersion parameter for binomial family taken to be 1)

   Null deviance: 14.17845  on 11  degrees of freedom
Residual deviance:  0.36479  on 10  degrees of freedom
AIC: 4.8107

Number of Fisher Scoring iterations: 12


$ld.est
            LD    SE    LCL     UCL
p = 0.50: 1156.918 1.131 909.032 1472.400
p = 0.90: 1250.719 1.145 958.940 1631.278
p = 0.95: 1357.561 1.345 759.505 2426.545

$comparison
[1] 0.9999986

$R2_Naglekerke
[1] 0.974
```

****
Cyt1Aa D11N Solubilized DTT
****

```
   probit    logit   cloglog   cauchit
1 1.106634 0.7100102 0.4655829 0.3349157

$Resum

Call:
glm(formula = x ~ log(d), family = family(i), data = status)

Deviance Residuals:
    Min       1Q   Median       3Q      Max
-0.23003  -0.06406   0.09788   0.19581   0.25164

Coefficients:
            Estimate Std. Error z value Pr(>|z|)
(Intercept)  -13.346     11.807  -1.130    0.258
log(d)         2.080      1.852   1.123    0.261

(Dispersion parameter for binomial family taken to be 1)

    Null deviance: 7.26574  on 12  degrees of freedom
Residual deviance: 0.33492  on 11  degrees of freedom
AIC: 10.217

Number of Fisher Scoring iterations: 7


$ld.est
              LD     SE     LCL         UCL
p = 0.50:   610.702  1.558 256.123    1456.165
p = 0.90:  2680.923  4.217 159.722   44999.141
p = 0.95: 12699.942 16.278  53.591 3009610.833

$comparison
[1] 0.9999998

$R2_Naglekerke
[1] 0.954
```

****
Cyt1Aa E32Q Solubilized DTT
****

```
   probit    logit   cloglog   cauchit
1 1.334224 0.9340985 0.7723857 0.1940535
```

```
$Resum

Call:
glm(formula = x ~ log(d), family = family(i), data = status)

Deviance Residuals:
    Min       1Q   Median       3Q      Max
-0.07921  -0.03132   0.07095   0.15492   0.28159

Coefficients:
          Estimate Std. Error z value Pr(>|z|)
(Intercept)  -30.236     31.140  -0.971    0.332
log(d)         3.683      3.766   0.978    0.328

(Dispersion parameter for binomial family taken to be 1)

    Null deviance: 7.97730  on 10  degrees of freedom
Residual deviance: 0.19405  on  9  degrees of freedom
AIC: 6.8412

Number of Fisher Scoring iterations: 8


$ld.est
             LD    SE     LCL       UCL
p = 0.50:  3675.988 1.530 1596.549   8463.811
p = 0.90:  8477.885 2.482 1427.296  50357.125
p = 0.95: 20411.609 5.783  654.752 636323.316

$comparison
[1] 0.9999995

$R2_Naglekerke
[1] 0.976


****************************
Cyt1Aa Y171F Solubilized DTT
****************************

    probit     logit  cloglog   cauchit
1 1.307281 0.9309076 1.162064 0.1577064

$Resum

Call:
glm(formula = x ~ log(d), family = family(i), data = status)

Deviance Residuals:
    Min       1Q   Median       3Q      Max
-0.13174  -0.09264   0.02837   0.10841   0.25586
```

```
Coefficients:
        Estimate Std. Error z value Pr(>|z|)
(Intercept) -21.757    24.456  -0.890   0.374
log(d)        3.810     4.274   0.892   0.373

(Dispersion parameter for binomial family taken to be 1)

   Null deviance: 6.73215  on 9  degrees of freedom
Residual deviance: 0.15771  on 8  degrees of freedom
AIC: 7.5873

Number of Fisher Scoring iterations: 8


$ld.est
           LD    SE    LCL      UCL
p = 0.50:  301.887 1.373 162.073   562.316
p = 0.90:  677.070 2.598 104.253  4397.244
p = 0.95: 1582.977 6.553  39.747 63043.683

$comparison
[1] 0.9999985

$R2_Naglekerke
[1] 0.977
```

Supplementary Table 3: Effect of point Cyt1Aa and Cyt2Aa mutations performed in the present study and by others before us on crystal formation, solubility and toxin toxicity.

Mutation	Equivalent in Cyt1Aa	Change	Secondary structure	Crystal	Expression	Solubility	Toxicity					PC binding	Cry toxin binding		Synergism		Reference	Comment
							Bacteria	Mammalian cells	Mosquito cells	Mosquitoes	Other insects		Cry11Aa	Cry4Ba	Cry11Aa	Cry4Ba		
C7S		Polar neutral to polar neutral		Smaller	SAW	Increased		SAW				Yes (LUV)					This study	
D11N		Acidic charged to polar neutral		Bigger	SAW	SAW		SAW				Yes (LUV)					This study	
R25A		Basic charged to aliphatic		None	SAW			Reduced?	Reduced?	SAW							Ward *et al.* , 1988	Loss of cytotoxicity not clearly stated in the results but written in the Figure1
R30A		Basic charged to aliphatic		None	SAW			Reduced?	Reduced?	SAW							Ward *et al.* , 1988	Loss of cytotoxicity not clearly stated in the results but written in the Figure1
E32A		Acidic charged to aliphatic		SAW	SAW			SAW	SAW	SAW							Ward *et al.* , 1988	
E32Q		Acidic charged to polar neutral		SAW	Increased	SAW		SAW				Yes (LUV)					This study	
D33A		Acidic charged to aliphatic		SAW	SAW			SAW	SAW	SAW							Ward *et al.* , 1988	
E36A		Acidic charged to aliphatic		SAW	SAW			SAW	SAW	SAW							Ward *et al.* , 1988	
E45A		Acidic charged to aliphatic		SAW	SAW		Reduced	Reduced (8X)	Reduced (6-7X)	SAW		No					Ward *et al.* , 1988	
E45N		Acidic charged to polar neutral		SAW	SAW			SAW	SAW	SAW							Ward *et al.* , 1988	
E45Q		Acidic charged to polar neutral		Bigger	SAW	SAW		Reduced (14X)				No (LUV)					This study	
D47A		Acidic charged to aliphatic		SAW	SAW			SAW	SAW	SAW							Ward *et al.* , 1988	
L58E		Aliphatic to acidic charged		SAW	Reduced	Insoluble											Bravo *et al.* , 2018	Data not shown for solubility
A59C		Aliphatic to polar neutral		SAW	SAW			Not toxic		Reduced (2X)	Increased (5X)	Yes (SUV)					Bravo *et al.* , 2018	
A59E		Aliphatic to acidic charged		None	None												Bravo *et al.* , 2018	
A61C		Aliphatic to polar neutral		SAW	SAW			Reduced		Increased (5X)	Increased (11X)	Yes (SUV)					Bravo *et al.* , 2018	
F62R		Aromatic to basic charged		None	None												Bravo *et al.* , 2018	
D72A		Acidic charged to aliphatic		SAW	SAW			SAW	SAW	SAW							Ward *et al.* , 1988	
D75A		Acidic charged to aliphatic		SAW	SAW			SAW	SAW	SAW							Ward *et al.* , 1988	
R78A		Basic charged to aliphatic		Smaller	Reduced					Reduced							Ward *et al.* , 1988	
K83A		Basic charged to aliphatic		SAW	SAW			SAW	SAW	SAW							Ward *et al.* , 1988	
E86A		Acidic charged to aliphatic		SAW	SAW			SAW	SAW	SAW							Ward *et al.* , 1988	
D102A		Acidic charged to aliphatic		SAW	SAW			SAW	SAW	SAW							Ward *et al.* , 1988	
M115T		Aliphatic to polar neutral					Reduced										Zghal *et al.* , 2018	
I116E		Aliphatic to acidic charged		Smaller													Lopez-Diaz et al., 2013	
K118A		Basic charged to aliphatic		SAW	SAW			SAW	SAW	SAW							Ward *et al.* , 1988	
V119E		Aliphatic to acidic charged		Smaller													Lopez-Diaz et al., 2013	
L120K		Aliphatic to basic charged		SAW				SAW		Increased (2X)		Yes (SUV)			Increased		Lopez-Diaz *et al.* , 2013; Bravo *et al.* , 2018	
E121A		Acidic charged to aliphatic		SAW	SAW			SAW	SAW	SAW							Ward *et al.* , 1988	
V122E		Aliphatic to acidic charged		SAW				Not toxic		Reduced (>9X)		No (SUV)			Increased		Lopez-Diaz *et al.* , 2013; Bravo *et al.* , 2018	
L123K		Aliphatic to basic charged		SAW				SAW		Increased (3X)		Yes (SUV)			Increased		Lopez-Diaz *et al.* , 2013; Bravo *et al.* , 2018	
K124A		Basic charged to aliphatic		Smaller	Increased			Increased (4X)	Increased (3-4X)	Increased (3X)		Yes					Ward *et al.* , 1988	
V126E		Aliphatic to acidic charged		SAW				Reduced		Reduced (>9X)		No (SUV)			Increased		Lopez-Diaz *et al.* , 2013; Bravo *et al.* , 2018	
D137A		Acidic charged to aliphatic		SAW	SAW			SAW	SAW	SAW							Ward *et al.* , 1988	
K154A		Basic charged to aliphatic		SAW	SAW			Not toxic	Reduced (14-18X)	Reduced (>8X)		No					Ward *et al.* , 1988	
K154R		Basic charged to basic charged		SAW	SAW			SAW	SAW	SAW							Ward *et al.* , 1988	
E156A		Acidic charged to aliphatic		SAW	SAW			SAW	SAW	SAW							Ward *et al.* , 1988	
K163A		Basic charged to aliphatic		SAW	SAW			Not toxic	Reduced (4-6X)	Reduced (4X)		No					Ward *et al.* , 1988	
K163R		Basic charged to basic charged		SAW	SAW			SAW	SAW	SAW							Ward *et al.* , 1988	
E164A		Acidic charged to aliphatic		SAW	SAW			Reduced (2X)	Reduced (4-7X)	Reduced (4X)		No					Ward *et al.* , 1988	
Q168E		Polar neutral to acidic charged		Longer	Reduced	Increased		Not toxic				No (LUV)					This study	
Y171F		Aromatic to aromatic		Smaller	Reduced	SAW		SAW				Yes (LUV)					This study	
C190V		Polar neutral to aliphatic		Smaller	SAW	SAW		SAW				Yes (LUV)					This study	
E196A		Acidic charged to aliphatic		SAW	SAW			SAW	SAW	SAW							Ward *et al.* , 1988	
K198A		Basic charged to aliphatic		SAW	SAW			SAW	SAW	SAW			SAW	Reduced (2X)	Increased (2X)	Reduced (3X)	Ward *et al.* , 1988; Perez *et al.* , 2005; Perez et al., 2007; Canton *et al.* , 2011	
K203A		Basic charged to aliphatic		Smaller	Increased					Reduced							Ward *et al.* , 1988	
E204A		Acidic charged to aliphatic		Smaller	Increased			Reduced (4X)	Reduced (3-14X)	Reduced (>8X)		No	Reduced (4X)	Reduced (2X)	Reduced (2X)	Reduced (3X)	Ward *et al.* , 1988; Perez *et al.* , 2005; Canton *et al.* , 2011	
D213A		Acidic charged to aliphatic		SAW	SAW			SAW	Reduced (8-14X)	Reduced (8X)		No					Ward *et al.* , 1988	
K225A		Basic charged to aliphatic		SAW	SAW			Not toxic	Reduced (>24X)	Reduced (>8X) / SAW		No	Reduced (10X)	Reduced (2X)	Reduced (5X)	Reduced (5X)	Ward *et al.* , 1988; Perez *et al.* , 2005; Perez et al., 2007; Canton *et al.* , 2011	Inconsistency for mosquito activity between studies
D240A		Acidic charged to aliphatic		Smaller	Increased					Reduced							Ward *et al.* , 1988	

SAW = Same As WT

Red = Cyt1Aa features affected by mutation

In Cyt2Aa toxin

R25A	T23A	Basic charged to aliphatic			SAW	SAW		SAW		SAW							Thammachat *et al.* , 2010	R25 in Cyt2Aa2 not conserved (replaced by T23 in Cyt1Aa)
P27A	R25A	Special to aliphatic			SAW	SAW		SAW		SAW							Thammachat *et al.* , 2010	P27 in Cyt2Aa2 not conserved (replaced by R25 in Cyt1Aa)
I31A	L29A	Aliphatic to aliphatic			SAW	SAW		SAW		SAW							Thammachat *et al.* , 2010	I31 in Cyt2Aa2 (= L29 in Cyt1Aa) - Conserved aminoacid
L33A	V31A	Aliphatic to aliphatic			Reduced	SAW		Reduced (6-130X)		Reduced (46X) in Aae							Thammachat *et al.* , 2010	L33 in Cyt2Aa2 (= V31 in Cyt1Aa) - Conserved aminoacid
A57L	A55L	Aliphatic to aliphatic			SAW	Insoluble				Reduced (230X) in Cqu / Not toxic in Aae							Promdonkoy *et al.* , 2008	A57 in Cyt2Aa2 (= A55L in Cyt1Aa) - Highly conserved aminoacid
A61C	A59C	Aliphatic to polar neutral			SAW	SAW		Reduced / Not toxic		SAW		No					Promdonkoy *et al.* , 2008; Promdonkoy & Ellar, 2000	A61 in Cyt2Aa2 (= A59C in Cyt1Aa) - Conserved aminoacid
S108C	S113C	Polar neutral to polar neutral			SAW	SAW		Reduced		Reduced (54X) in Cqu / Reduced (4X) in Aae		Reduced					Promdonkoy *et al.* , 2008	C108 in Cyt2Aa2 (= S113C in Cyt1Aa) - Highly conserved aminoacid
V109A	V114A	Aliphatic to aliphatic			SAW	SAW		SAW		Reduced (180X) in Cqu / Reduced (4X) in Aae		Yes					Promdonkoy *et al.* , 2008	V109 in Cyt2Aa2 (= V114A in Cyt1Aa) - Conserved aminoacid
M110A	M115A	Aliphatic to aliphatic			SAW	Reduced				Not toxic							Promdonkoy *et al.* , 2008	M110 in Cyt2Aa2 (= M115A in Cyt1Aa) - Highly conserved aminoacid ; unstable toxin
V111C	I116C	Aliphatic to polar neutral		SAW	SAW			Not toxic									Promdonkoy & Ellar, 2000	V111 in Cyt2Aa1 (= I116 in Cyt1Aa) - Conserved between Cyt1A, Cyt1B, Cyt2B but not Cyt2A
L114A	V119A	Aliphatic to aliphatic			SAW	Reduced				Reduced (92X) in Cqu / Not toxic in Aae							Promdonkoy *et al.* , 2008	L114 in Cyt2Aa2 not conserved (replaced by V119 in Cyt1Aa) ; unstable toxin
I118A	L123A	Aliphatic to aliphatic			SAW	Reduced				Reduced (108X) in Cqu / Not toxic in Aae							Promdonkoy *et al.* , 2008	I118 in Cyt2Aa2 not conserved (replaced by L123 in Cyt1Aa) ; unstable toxin
W132F	I136F	Aromatic to aromatic		SAW	SAW	Insoluble				Not toxic in Cqu & Aae							Promdonkoy *et al.* , 2004	W132 in Cyt2Aa2 not conserved (replaced by I136 in Cyt1Aa)
W132A	I136A	Aromatic to aliphatic				Reduced				Not toxic in Cqu & Aae							Promdonkoy *et al.* , 2004	W132 in Cyt2Aa2 not conserved (replaced by I136 in Cyt1Aa)
W132D	I136D	Aromatic to acidic charged				Reduced				Not toxic in Cqu & Aae							Promdonkoy *et al.* , 2004	W132 in Cyt2Aa2 not conserved (replaced by I136 in Cyt1Aa)
W132E	I136E	Aromatic to acidic charged				Reduced				Not toxic in Cqu & Aae							Promdonkoy *et al.* , 2004	W132 in Cyt2Aa2 not conserved (replaced by I136 in Cyt1Aa)
W132F	I136F	Aromatic to aromatic				Reduced				Not toxic in Cqu & Aae							Promdonkoy *et al.* , 2004	W132 in Cyt2Aa2 not conserved (replaced by I136 in Cyt1Aa)
W132G	I136G	Aromatic to special				Reduced				Not toxic in Cqu & Aae							Promdonkoy *et al.* , 2004	W132 in Cyt2Aa2 not conserved (replaced by I136 in Cyt1Aa)
W132I	I136I	Aromatic to aliphatic				Reduced				Not toxic in Cqu & Aae							Promdonkoy *et al.* , 2004	W132 in Cyt2Aa2 not conserved (replaced by I136 in Cyt1Aa)
W132K	I136K	Aromatic to basic charged				Reduced				Not toxic in Cqu & Aae							Promdonkoy *et al.* , 2004	W132 in Cyt2Aa2 not conserved (replaced by I136 in Cyt1Aa)
W132L	I136L	Aromatic to aliphatic				Reduced				Not toxic in Cqu & Aae							Promdonkoy *et al.* , 2004	W132 in Cyt2Aa2 not conserved (replaced by I136 in Cyt1Aa)
W132M	I136M	Aromatic to aliphatic				Reduced				Not toxic in Cqu & Aae							Promdonkoy *et al.* , 2004	W132 in Cyt2Aa2 not conserved (replaced by I136 in Cyt1Aa)
W132N	I136N	Aromatic to polar neutral				Reduced				Not toxic in Cqu & Aae							Promdonkoy *et al.* , 2004	W132 in Cyt2Aa2 not conserved (replaced by I136 in Cyt1Aa)
W132Q	I136Q	Aromatic to polar neutral				SAW				Not toxic in Cqu & Aae							Promdonkoy *et al.* , 2004	W132 in Cyt2Aa2 not conserved (replaced by I136 in Cyt1Aa)
W132R	I136R	Aromatic to basic charged				SAW				Not toxic in Cqu & Aae							Promdonkoy *et al.* , 2004	W132 in Cyt2Aa2 not conserved (replaced by I136 in Cyt1Aa)
W132S	I136S	Aromatic to polar neutral				Reduced				Not toxic in Cqu & Aae							Promdonkoy *et al.* , 2004	W132 in Cyt2Aa2 not conserved (replaced by I136 in Cyt1Aa)
W132T	I136T	Aromatic to polar neutral				Reduced				Not toxic in Cqu & Aae							Promdonkoy *et al.* , 2004	W132 in Cyt2Aa2 not conserved (replaced by I136 in Cyt1Aa)
W132V	I136V	Aromatic to aliphatic				Reduced				Not toxic in Cqu & Aae							Promdonkoy *et al.* , 2004	W132 in Cyt2Aa2 not conserved (replaced by I136 in Cyt1Aa)
T144A	T148A	Polar neutral to aliphatic		SAW	SAW	SAW		Reduced (2X)		SAW		Yes					Suktham *et al.* , 2013	T144 in Cyt2Aa2 (=T148A in Cyt1Aa) - Highly conserved aminoacid
N145A	N149A	Polar neutral to aliphatic		SAW	SAW	SAW		Not toxic		Not toxic		No					Suktham *et al.* , 2013	N145 in Cyt2Aa2 (=N149A in Cyt1Aa) - Highly conserved aminoacid
I150A	K154A	Aliphatic to aliphatic			SAW	SAW		Not toxic		Not toxic		No					Pathaichindachote *et al.* , 2013; Promdonkoy & Ellar, 2005	I150 in Cyt2Aa1 & Cyt2Aa2 not conserved (replaced by K154 in Cyt1Aa)
I150K	*K154K*	Aliphatic to basic charged			SAW	SAW		Reduced (20X)		Increased (7X) in Cqu / Not toxic in Aae		No					Pathaichindachote *et al.* , 2013	I150 in Cyt2Aa2 not conserved (replaced by K154 in Cyt1Aa)
I150F	K154F	Aliphatic to aromatic			SAW	SAW		Not toxic		Not toxic		No					Pathaichindachote *et al.* , 2013	I150 in Cyt2Aa2 not conserved (replaced by K154 in Cyt1Aa)
I150L	K154L	Aliphatic to aliphatic			SAW	SAW		SAW		SAW in Cqu / Reduced (3X) in Aae		Yes					Pathaichindachote *et al.* , 2013	I150 in Cyt2Aa2 not conserved (replaced by K154 in Cyt1Aa)
I150E	K154E	Aliphatic to acidic charged			SAW	SAW		SAW		SAW in Cqu / Reduced (2X) in Aae		Yes					Pathaichindachote *et al.* , 2013	I150 in Cyt2Aa2 not conserved (replaced by K154 in Cyt1Aa)
W154F	W158F	Aromatic to aromatic		SAW	SAW	Reduced		SAW		Not toxic in Cqu & Aae							Promdonkoy *et al.* , 2004	W154 in Cyt2Aa2 (=W158 in Cyt1Aa) - Highly conserved aminoacid
W154A	W158A	Aromatic to aliphatic				Reduced				Not toxic in Cqu & Aae							Promdonkoy *et al.* , 2004	W154 in Cyt2Aa2 (=W158 in Cyt1Aa) - Highly conserved aminoacid
W154C	W158C	Aromatic to polar neutral				Reduced				Not toxic in Cqu & Aae							Promdonkoy *et al.* , 2004	W154 in Cyt2Aa2 (=W158 in Cyt1Aa) - Highly conserved aminoacid
W154D	W158D	Aromatic to acidic charged				Reduced				Not toxic in Cqu & Aae							Promdonkoy *et al.* , 2004	W154 in Cyt2Aa2 (=W158 in Cyt1Aa) - Highly conserved aminoacid
W154F	W158F	Aromatic to aromatic				Reduced				Not toxic in Cqu & Aae							Promdonkoy *et al.* , 2004	W154 in Cyt2Aa2 (=W158 in Cyt1Aa) - Highly conserved aminoacid
W154G	W158G	Aromatic to special				Reduced				Not toxic in Cqu & Aae							Promdonkoy *et al.* , 2004	W154 in Cyt2Aa2 (=W158 in Cyt1Aa) - Highly conserved aminoacid
W154H	W158H	Aromatic to basic charged				Reduced				Not toxic in Cqu & Aae							Promdonkoy *et al.* , 2004	W154 in Cyt2Aa2 (=W158 in Cyt1Aa) - Highly conserved aminoacid
W154K	W158K	Aromatic to basic charged				Reduced				Not toxic in Cqu & Aae							Promdonkoy *et al.* , 2004	W154 in Cyt2Aa2 (=W158 in Cyt1Aa) - Highly conserved aminoacid
W154L	W158L	Aromatic to aliphatic				Reduced				Not toxic in Cqu & Aae							Promdonkoy *et al.* , 2004	W154 in Cyt2Aa2 (=W158 in Cyt1Aa) - Highly conserved aminoacid
W154P	W158P	Aromatic to special				Reduced				Not toxic in Cqu & Aae							Promdonkoy *et al.* , 2004	W154 in Cyt2Aa2 (=W158 in Cyt1Aa) - Highly conserved aminoacid
W154Q	W158Q	Aromatic to polar neutral				Reduced				Not toxic in Cqu & Aae							Promdonkoy *et al.* , 2004	W154 in Cyt2Aa2 (=W158 in Cyt1Aa) - Highly conserved aminoacid
W154R	W158R	Aromatic to basic charged				Reduced				Not toxic in Cqu & Aae							Promdonkoy *et al.* , 2004	W154 in Cyt2Aa2 (=W158 in Cyt1Aa) - Highly conserved aminoacid
W154S	W158S	Aromatic to polar neutral				Reduced				Not toxic in Cqu & Aae							Promdonkoy *et al.* , 2004	W154 in Cyt2Aa2 (=W158 in Cyt1Aa) - Highly conserved aminoacid
W154T	W158T	Aromatic to polar neutral				Reduced				Not toxic in Cqu & Aae							Promdonkoy *et al.* , 2004	W154 in Cyt2Aa2 (=W158 in Cyt1Aa) - Highly conserved aminoacid
W154Y	W158Y	Aromatic to aromatic				Reduced				Not toxic in Cqu & Aae							Promdonkoy *et al.* , 2004	W154 in Cyt2Aa2 (=W158 in Cyt1Aa) - Highly conserved aminoacid
W157F	W161F	Aromatic to aromatic		SAW	SAW	SAW		SAW		SAW in Cqu & Aae							Promdonkoy *et al.* , 2004	W157 in Cyt2Aa2 (=W161 in Cyt1Aa) - Highly conserved aminoacid

W157V	W161V	Aromatic to hydrophobic			Reduced		Not toxic in Cqu & Aae	Promdonkoy *et al.* , 2004	W157 in Cyt2Aa2 (=W161 in Cyt1Aa) - Highly conserved aminoacid
S166C	N170C	Polar neutral to polar neutral	SAW	SAW		Not toxic		Promdonkoy & Ellar, 2000	S166 in Cyt2Aa1 not conserved (replaced by N170 in Cyt1Aa)
Y169C	Y173C	Aromatic to polar neutral	None	Reduced				Promdonkoy & Ellar, 2000	Y169 in Cyt2Aa1 (=Y173 in Cyt1Aa) - Highly conserved aminoacid
L172C	L176C	Aliphatic to polar neutral	SAW	SAW		Reduced (16X)		Promdonkoy & Ellar, 2000	L172 in Cyt2Aa1 (=L176 in Cyt1Aa) - Conserved aminoacid
V186C	*C190C*	Aliphatic to polar neutral	SAW	SAW		SAW		Promdonkoy & Ellar, 2000	V186 in Cyt2Aa1 not conserved (replaced by C190 in Cyt1Aa)
L189C	V193C	Aliphatic to polar neutral	SAW	SAW		Reduced (128X)		Promdonkoy & Ellar, 2000	L189 in Cyt2Aa1 not conserved (replaced by V193 in Cyt1Aa)
E192C	E196C	Acidic charged to polar neutral	SAW	SAW		Not toxic		Promdonkoy & Ellar, 2000	E192 in Cyt2Aa1 (=E196 in Cyt1Aa) - Highly conserved aminoacid
V202C	V206C	Aliphatic to polar neutral	SAW	SAW		Not toxic		Promdonkoy & Ellar, 2000	V202 in Cyt2Aa1 (=V206 in Cyt1Aa) - Conserved aminoacid
E214C	N218C	Acidic charged to polar neutral	SAW	SAW		Reduced (4X)		Promdonkoy & Ellar, 2000	E214 in Cyt2Aa1 not conserved (replaced by N218 in Cyt1Aa)
M217C	I221C	Aliphatic to polar neutral	SAW	SAW		Not toxic		Promdonkoy & Ellar, 2000	M217 in Cyt2Aa1 not conserved (replaced by I221 in Cyt1Aa)
L220C	L224C	Aliphatic to polar neutral	SAW	SAW		SAW		Promdonkoy & Ellar, 2000	L220 in Cyt2Aa1 (=L224 in Cyt1Aa) - Highly conserved aminoacid
T221A	K225A	Polar neutral to polar neutral		SAW	SAW	SAW	SAW	Promdonkoy & Ellar, 2005	T221 in Cyt2Aa2 not conserved (replaced by K225 in Cyt1Aa)
V223C	A227C	Aliphatic to polar neutral	SAW	SAW		Reduced (256X)		Promdonkoy & Ellar, 2000	V223 in Cyt2Aa1 not conserved (replaced by A227 in Cyt1Aa)
L226C	L230C	Aliphatic to polar neutral	SAW	SAW		Reduced (256X)		Promdonkoy & Ellar, 2000	L226 in Cyt2Aa1 (= L230 in Cyt1Aa) - Highly conserved aminoacid
N230A	S234A	Polar neutral to aliphatic		Reduced	Reduced	Reduced (1-4.6X)	Reduced (15X) in Aae	Thammachat *et al.* , 2010	N230 in Cyt2Aa2 (=S234 in Cyt1Aa) - Conserved aminoacid
I233A	I238A	Aliphatic to aliphatic		SAW	Reduced	Reduced (3-27X)	SAW	Thammachat *et al.* , 2010	I233 in Cyt2Aa2 (=I238 in Cyt1Aa) - Highly conserved aminoacid
I233Stop	I238Stop	Aliphatic to stop		Reduced	Reduced	Reduced (3-520X)	Reduced (24X) in Aae	Thammachat *et al.* , 2010	I233 in Cyt2Aa2 (=I238 in Cyt1Aa) - Highly conserved aminoacid

Supplementary Table 4. List of all primers used for plasmid construction.

Mutation	Name (Primer)	Forward/ Reverse	Primer sequence (5'-3')	Length (Primer)	Tm (Primer)	Backbone	Notes
/	Amp_F1	Forward	GCCGCAGTGTTATCACTCATGGTTATGGC	29	64.0	pWF45	
C7S	Cyt1A-C7S_R1m	Reverse	tcttctaatgga**G**aatgatttaaattttccataaataaacaactcc	46	60.0	pWF45	
C190V	Cyt1A-C190V_R1m	Reverse	ctggtaca**AC**atacataacgccaccagtttg	46	63.0	pWF45	Amp_F1 was used with each reverse primer of Cyt1Aa mutant construction. For each mutation primer, the inserted mutation is indicated as a capital bold letter. To generate the vector containing the mutation, the two fragments generated for the same mutations were assembled by Gibson assembly following the procedure described in the manuscript.
D11N	Cyt1A-D11N_R1m	Reverse	cctttatat**T**ttctaatggacaatgatttaaattttccat	46	58.0	pWF45	
E32Q	Cyt1A-E32Q_R1m	Reverse	ggatcct**G**aacacgtaatgtaataacccttgc	46	62.0	pWF45	
E45Q	Cyt1A-E45Q_R1m	Reverse	atcaattt**G**gttaatagaaagaagattattgatttcatttgg	46	59.0	pWF45	
Q168E	Cyt1A-Q168E_R1m	Reverse	gtatgtgtaatttgttt**C**attagcagtttccttg	46	59.0	pWF45	
Y171F	Cyt1A-Y171F_R1m	Reverse	gtatgtg**A**aatttgtttgattagcagtttccttgc	46	60.0	pWF45	
/	Amp_R1	Reverse	AGTGCTGCCATAACCATGAGTGATAACACT	30	63.0	pWF45	
C7S	Cyt1A-C7S_F1m	Forward	catt**C**tccattagaagatataaaggtaaatccatgg	46	59.0	pWF45	
C190V	Cyt1A-C190V_F1m	Forward	cgttatgtat**GT**tgtaccagttggttttgaaatt	46	60.0	pWF45	Amp_R1 was used with each forward primer of Cyt1Aa mutant construction. For each mutation primer, the inserted mutation is indicated as a capital bold letter. To generate the vector containing the mutation, the two fragments generated for the same mutations were assembled by Gibson assembly following the procedure described in the manuscript.
D11N	Cyt1A-D11N_F1m	Forward	cattgtccattagaa**A**atataaaggtaaatccatgg	46	59.0	pWF45	
E32Q	Cyt1A-E32Q_F1m	Forward	cattacgtgtt**C**aggatccaaatgaaatcaataatc	46	60.0	pWF45	
E45Q	Cyt1A-E45Q_F1m	Forward	ctattaac**C**aaattgataatccgaattatatattgcaagc	46	59.0	pWF45	
Q168E	Cyt1A-Q168E_F1m	Forward	ctgctaat**G**aaacaaattacacatacaatgtcctg	46	60.0	pWF45	
Y171F	Cyt1A-Y171F_F1m	Forward	caaacaaatt**T**cacatacaatgtcctgtttgc	46	60.0	pWF45	
