# Supplementary material for: Serial femtosecond crystallography on in vivo-grown crystals drives elucidation of mosquitocidal Cyt1Aa bioactivation cascade
Source: Nat Commun. 2020 Mar 2;11:1153. doi: 10.1038/s41467-020-14894-w (PMC7052140; doi:10.1038/s41467-020-14894-w)
Supplement: Supplementary file 3 — Description of Additional Supplementary Files [file 41467_2020_14894_MOESM3_ESM.pdf]

Description of Additional Supplementary Files

File Name: Supplementary Movie 1
Description: Sf21 cell exposed to 1.4 nm FITC-Dextran only (control).

File Name: Supplementary Movie 2
Description: Sf21 cell exposed simultaneously to 1.4 nm FITC-Dextran and to 400 nM of Cyt1Aa activated toxin.

File Name: Supplementary Movie 3
Description: NIH cell exposed to 1.4 nm FITC-Dextran only (control).

File Name: Supplementary Movie 4
Description: NIH cell exposed simultaneously to 1.4 nm FITC-Dextran and to 400 nM of Cyt1Aa activated toxin.
