# Supplementary material for: Serial femtosecond crystallography on in vivo-grown crystals drives elucidation of mosquitocidal Cyt1Aa bioactivation cascade
Source: Nat Commun. 2020 Mar 2;11:1153. doi: 10.1038/s41467-020-14894-w (PMC7052140; doi:10.1038/s41467-020-14894-w)
Supplement: Supplementary file 8 — Reporting Summary [file 41467_2020_14894_MOESM8_ESM.pdf]

natureresearch

Corresponding author(s): Dr. Jacques-Philippe Colletier

Last updated by author(s): Jan 23, 2020

Reporting Summary

Nature Research wishes to improve the reproducibility of the work that we publish. This form provides structure for consistency and transparency in reporting. For further information on Nature Research policies, see Authors & Referees and the Editorial Policy Checklist .

Statistics

For all statistical analyses, confirm that the following items are present in the figure legend, table legend, main text, or Methods section.

n/a	Confirmed	
☐	☒	The exact sample size (*n*) for each experimental group/condition, given as a discrete number and unit of measurement
☐	☒	A statement on whether measurements were taken from distinct samples or whether the same sample was measured repeatedly
☐	☒	The statistical test(s) used AND whether they are one- or two-sided<br>*Only common tests should be described solely by name; describe more complex techniques in the Methods section.*
☒	☐	A description of all covariates tested
☐	☒	A description of any assumptions or corrections, such as tests of normality and adjustment for multiple comparisons
☐	☒	A full description of the statistical parameters including central tendency (e.g. means) or other basic estimates (e.g. regression coefficient) AND variation (e.g. standard deviation) or associated estimates of uncertainty (e.g. confidence intervals)
☐	☒	For null hypothesis testing, the test statistic (e.g. *F*, *t*, *r*) with confidence intervals, effect sizes, degrees of freedom and *P* value noted<br>*Give P values as exact values whenever suitable.*
☒	☐	For Bayesian analysis, information on the choice of priors and Markov chain Monte Carlo settings
☒	☐	For hierarchical and complex designs, identification of the appropriate level for tests and full reporting of outcomes
☒	☐	Estimates of effect sizes (e.g. Cohen's *d*, Pearson's *r*), indicating how they were calculated

Our web collection on statistics for biologists contains articles on many of the points above.

Software and code

Policy information about availability of computer code

Data collection	Scanning electron micrographs (SEM) were acquired using a Zeiss LEO 1530 microscope and the associated commercial software.<br>Atomic force microscopy (AFM) images were acquired using the NanoScope software.<br>Protein gels were digitalized using a ChemiDoc XRS+ imaging system piloted by the Image Lab software version 6.0.0.<br>FACS raw data were collected, treated and processed using the MACSQuantify software v2.11.<br>For exposure of cells to FITC-dextran beads, cell images were acquired using the commercial software associated with the laser scanning confocal microscope (LSM), Zeiss LSM 510.<br>Black lipid membrane (BLM) data were recorded using an Axon Digidata 1440A digitizer and associated commercial software.<br>Transmission electron micrographs (TEM) were acquired using the commercial software associated with the FEI Tecnai T12 and FEI F20 microscopes.
Data analysis	All collected diffraction pattern images were indexed with DIALS (ref. 37), using the cctbx.xfel graphical user interface. Data were merged using cxi.merge. pH7 dataset was phased by molecular replacement with Phaser. Further refinement in real space was performed using Coot, and refinement in the reciprocal space using Refmac. Fo-Fo maps were q-weighted and produced using a CNS custom-written script (all scripts and methods available in refs 43 to 45).<br>ImageJ v1.51k was used to determine crystal sizes (SEM), diameters and surfaces of pores (AFM), and to evaluate the size of proteins present in SDS-PAGE bands. AFM Images were processed with Gwyddion 35 and, when needed, stripe noise was removed using DeStripe. MALDI data were processed with Flexanalysis 3.0 software. BLM data analysis was performed with clampfit.<br>The software R 3.5.2 was used to calculate all statistical indicators. pH solubility and mortalities were analyzed using a modified code from Savi et al. 2018 (binomial model selection and SP and LC measurements).

For manuscripts utilizing custom algorithms or software that are central to the research but not yet described in published literature, software must be made available to editors/reviewers. We strongly encourage code deposition in a community repository (e.g. GitHub). See the Nature Research guidelines for submitting code & software for further information.

Data

Policy information about availability of data

All manuscripts must include a data availability statement. This statement should provide the following information, where applicable:

- Accession codes, unique identifiers, or web links for publicly available datasets
- A list of figures that have associated raw data
- A description of any restrictions on data availability

Structures and structure factor amplitudes have been deposited in the PDB databank under accession codes 6T14 (‘pH7’; [10.2210/pdb6T14/pdb]), 6T19 (‘DTT’; [10.2210/pdb6T19/pdb]), 6T1A (‘pH10’; [10.2210/pdb6T1A/pdb]) and 6T1C (‘C7S mutant’; [10.2210/pdb6T1C/pdb]). The source data underlying Figs. 3, 4a-g, 5d-h and 5j-q and Supplementary Figs. 5, 7-12 and 14b are provided as a Source Data file. Other data are available from the corresponding author upon reasonable request.

Field-specific reporting

Please select the one below that is the best fit for your research. If you are not sure, read the appropriate sections before making your selection.

☒ Life sciences ☐ Behavioural & social sciences ☐ Ecological, evolutionary & environmental sciences

For a reference copy of the document with all sections, see nature.com/documents/nr-reporting-summary-flat.pdf

Life sciences study design

All studies must disclose on these points even when the disclosure is negative.

Sample size	Sample size was selected based on the sample size for each type of analysis accepted from the peer-reviewed litterature.
Data exclusions	No data were excluded from analysis.
Replication	All experiments were repeated at least twice to ensure their reproducibility and the biological relevance of the findings. The replication information is indicated in the manuscript for each experiment when relevant.
Randomization	When testing different concentrations and different samples, measurements were performed randomly (i.e. different measures for the same sample were not performed in a row).
Blinding	Sample preparation was disconnected from data measurement and performed by two different persons when possible. Samples were attributed code names and measured without reference to their identity. Only after data collection, anonymity was lifted for data analysis.

Reporting for specific materials, systems and methods

We require information from authors about some types of materials, experimental systems and methods used in many studies. Here, indicate whether each material, system or method listed is relevant to your study. If you are not sure if a list item applies to your research, read the appropriate section before selecting a response.

Materials & experimental systems

n/a	Involved in the study	
☒	☐	Antibodies
☐	☒	Eukaryotic cell lines
☒	☐	Palaeontology
☒	☐	Animals and other organisms
☒	☐	Human research participants
☒	☐	Clinical data

Methods

n/a	Involved in the study	
☒	☐	ChIP-seq
☐	☒	Flow cytometry
☒	☐	MRI-based neuroimaging

Eukaryotic cell lines

Policy information about cell lines

Cell line source(s)	Three cell lines were used in this study. Sf21 cells originating from Spodoptera frugiperda were obtained from, and authenticated by the American Type Culture Collection (ATCC; CRL-1711). NIH 3T3 cells originating from mouse fibroblast were also obtained from, and authenticated by ATCC (CRL-1658). HEK293 cells originating from human kidney embryonic cells were obtained from, and authenticated by Sigma Aldrich (85120602).
Authentication	All cells originate from commercially- available and unmodified cell lines. The latter were not further authenticated in the lab.
Mycoplasma contamination	Absence of mycoplasma contamination was confirmed before use for Sf21 and NIH 3T3 cell lines using the LookOut mycoplasma PCR Detection Kit (Sigma-Aldrich) and for HEK293 cells using the MycoAlert™ Mycoplasma Detection Kit (Lonza).
Commonly misidentified lines (See ICLAC register)	None

Flow Cytometry

Plots

Confirm that:

- [x] The axis labels state the marker and fluorochrome used (e.g. CD4-FITC).
- [x] The axis scales are clearly visible. Include numbers along axes only for bottom left plot of group (a 'group' is an analysis of identical markers).
- [x] All plots are contour plots with outliers or pseudocolor plots.
- [x] A numerical value for number of cells or percentage (with statistics) is provided.

Methodology

Sample preparation	We used flow cytometry to estimate the toxin-induced mortality on HEK293 cells. For each dose of the toxin (WT and mutants thereof) in each condition, a total of ~1 million cells suspended in 1 mL of FreeStyleTM 293 medium (Gibco) were incubated with the toxin at different concentrations and with 1 µg/mL of propidium iodide (PI) for 15 min.
Instrument	MACS Quant VYB FACS (Milenyi Biotec)
Software	MACSQuantify software v2.11
Cell population abundance	Purity of the cells were determined prior to FACS measurement by microscope observations. A mean of 27,092 (± 20,365; ± SD) cells were analyzed for each condition.
Gating strategy	Only one population of cells was analyzed. Therefore, a permissive preliminary gating was performed based on SSC/FSC to remove cell debris. This gating strategy is visible on the Supplementary Figure 14. No selection was performed based on FSC but only cells within 25-750 on the SSC scale were selected. Cells with a fluorescence value at 617 nm (channel B2 – built-in filter 589-639 nm range) higher than 8 counts were considered dead (= PI inserted into the cells) whereas cells with values lower than 8 were considered alive.

- [x] Tick this box to confirm that a figure exemplifying the gating strategy is provided in the Supplementary Information.
